# Hydrogen Peroxide Formation during Ozonation of Olefins and Phenol: Mechanistic Insights from Oxygen Isotope Signatures

**DOI:** 10.1021/acs.est.3c00788

**Published:** 2023-05-08

**Authors:** Joanna Houska, Laura Stocco, Thomas B. Hofstetter, Urs von Gunten

**Affiliations:** † Eawag Swiss Federal Institute of Aquatic Science and Technology, 8600 Dübendorf, Switzerland; ‡ School of Architecture, Civil, and Environmental Engineering, École Polytechnique Fédérale de Lausanne, 1015 Lausanne, Switzerland; § Department of Environmental System Science, ETH Zurich, 8092 Zurich, Switzerland

**Keywords:** ozonation, hydrogen peroxide, reaction mechanisms, olefins, phenol, oxygen isotopes, isotope ratio mass spectrometry

## Abstract

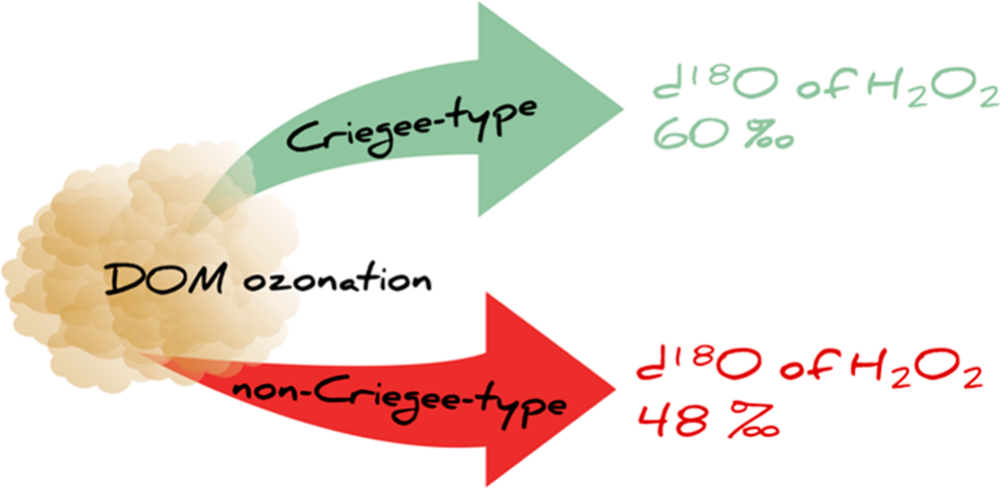

Mitigation of undesired byproducts from ozonation of dissolved organic matter (DOM) such as aldehydes and ketones is currently hampered by limited knowledge of their precursors and formation pathways. Here, the stable oxygen isotope composition of H_2_O_2_ formed simultaneously with these byproducts was studied to determine if it can reveal this missing information. A newly developed procedure, which quantitatively transforms H_2_O_2_ to O_2_ for subsequent ^18^O/^16^O ratio analysis, was used to determine the δ^18^O of H_2_O_2_ generated from ozonated model compounds (olefins and phenol, pH 3–8). A constant enrichment of ^18^O in H_2_O_2_ with a δ^18^O value of ∼59‰ implies that ^16^O–^16^O bonds are cleaved preferentially in the intermediate Criegee ozonide, which is commonly formed from olefins. H_2_O_2_ from the ozonation of acrylic acid and phenol at pH 7 resulted in lower ^18^O enrichment (δ^18^O = 47–49‰). For acrylic acid, enhancement of one of the two pathways followed by a carbonyl–H_2_O_2_ equilibrium was responsible for the smaller δ^18^O of H_2_O_2_. During phenol ozonation at pH 7, various competing reactions leading to H_2_O_2_ via an intermediate ozone adduct are hypothesized to cause lower δ^18^O in H_2_O_2_. These insights provide a first step toward supporting pH-dependent H_2_O_2_ precursor elucidation in DOM.

## Abstract 2

Oxygen isotope signatures in H_2_O_2_ provide novel mechanistic insights into its formation upon ozonation of olefins and phenol.

## Introduction

**Figure 1:**
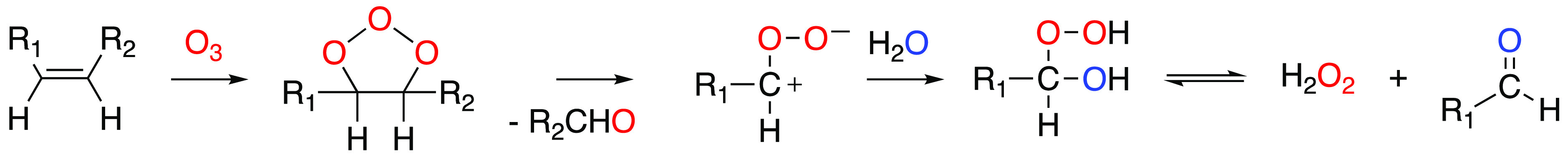
Criegee mechanism of a disubstituted olefin (−R_1_ and −R_2_) via the Criegee ozonide and the formation of two carbonyl compounds, an α-hydroxyalkylhydroperoxide and finally H_2_O_2_, which are in equilibrium. The oxygen atoms are colored based on their origin (red from O_3_ and blue from H_2_O).

Hydrogen peroxide (H_2_O_2_) is a common reactive oxygen species in natural and technical aquatic systems and in living organisms.^1−4^ During oxidative water treatment with ozone (O_3_), H_2_O_2_ is a secondary oxidant species which is formed via various reactions such as ozone self-decay and oxidation of organic compounds.^5−10^ One of the main formation pathways for H_2_O_2_ is the Criegee mechanism (Figure 1), where the sum of organic peroxides and H_2_O_2_ is formed with up to 100% yield (in % of consumed O_3_) along with potentially toxic aldehydes and ketones.^10−12^ Most of them are expected to be degraded during biological post-treatment.^13^

Aldehydes and ketones are formed from both phenols and olefins, but the H_2_O_2_ yields for phenols (∼18% at pH 7 and ∼36% at pH 3^5^) are generally much lower.^8,14^ For the ozonation of olefins, the stoichiometric formation of H_2_O_2_ is typically not pH-dependent.^10^ The pH dependence of the H_2_O_2_ yields from phenol could be related to multiple reaction pathways. H_2_O_2_ formation from phenol ozonation at pH 3 is mainly accompanied by the formation of organic acids, which points to a Criegee-type mechanism that proceeds in analogy to that shown in Figure 1.^5^ However, at pH 7, H_2_O_2_ formation is attributed to a combination of benzoquinone and organic acid formation, which involves reactions other than the Criegee mechanism.

Phenolic sites in dissolved organic matter (DOM) are generally considered the main oxidant-reactive groups, but olefinic moieties are also present at lower concentrations.^15−17^ Consequently, the formation of H_2_O_2_ upon ozonation of DOM is difficult to rationalize and the contribution of oxidant-reactive sites therein as well as the underlying formation pathways are not sufficiently understood. A previous study showed that both olefins and phenols form similar aldehydes and ketones during ozonation, but the two types of precursors from DOM can only be distinguished in rare cases.^12^ Since the same precursors lead to the formation of H_2_O_2_, a similar knowledge gap exists for H_2_O_2_.

Compound-specific isotope analysis (CSIA) offers complementary avenues to elucidate reaction mechanisms of organic chemicals during water treatment based on the evaluation of the natural abundance of the stable isotope composition of reaction products.^18−21^ Previous studies have used CSIA to study the formation of *N*-nitrosamines upon chloramination of various *N*-containing precursor compounds^22−24^ and have found that sequences of reactions and their isotope effects can lead to characteristic isotopic compositions. Upon chloramination, ^13^C/^12^C and ^15^N/^14^N ratios in *N*-nitrosamines are indicative of a specific formation pathway. Likewise, the observations of distinct ^13^C/^12^C ratios in chloroform formation during chlorination of DOM allowed distinguishing between resorcinol- and phenol-type precursors.^25^

Based on these findings, it is posited that the measurement of ^18^O/^16^O ratios of H_2_O_2_ generated in ozonation processes could reveal mechanistic information on the aforementioned reactions. The observation of different pathways to H_2_O_2_ from olefins and phenols and the different pH-dependent molar H_2_O_2_ yields^5,10^ may lead to pathway-dependent changes in δ^18^O of H_2_O_2_. As exemplified in Figure 1, the ozonation of olefins results in the transfer of only two of three O atoms of O_3_ to H_2_O_2_. A discrimination between reactions of heavy and light O atoms in O_3_ isotopologue intermediates that lead to H_2_O_2_ and other O-containing products can be expected. Therefore, partitioning of O atoms between H_2_O_2_ and other O-containing products could additionally contribute to fractionation in O isotopes in H_2_O_2_. However, O isotope fractionation of H_2_O_2_ has never been studied in the context of oxidative water treatment, partly because methods for δ^18^O quantification of H_2_O_2_ and O_3_ in aqueous matrices are unavailable.

Because the functioning of isotope ratio mass spectrometers requires the conversion of analytes into small analyte gases,^26^ H_2_O_2_ is oxidized to O_2_ for measurement of ^18^O/^16^O.^27−29^ This conversion has been achieved by three different methods: (i) conversion by catalase (H_2_O_2_ → 1/2 O_2_ + H_2_O),^23,28,30,31^ (ii) oxidation by permanganate in acidic solution (2 MnO_4_^–^ + 6 H^+^ + 5 H_2_O_2_ → 2 Mn^2+^ + 8 H_2_O + 5 O_2_),^32,27^ or (iii) oxidation by HOCl (HO_2_^–^ + HOCl → H_2_O + Cl^–^ + O_2_).^30^ The first method using catalase has been applied to determine the hydrogen and oxygen isotope composition of commercial H_2_O_2_,^28^ but only 50% of the H_2_O_2_ is transformed to O_2_, making it less favorable for experiments with low H_2_O_2_ yields. The second method using permanganate has been applied for determining δ^18^O in rainwater samples^27^ and in H_2_O_2_ self-decomposition experiments,^29^ but its application is hampered by the need of extensive extraction and purification procedures requiring several liters of sample. Moreover, it is unclear whether organic peroxides, which are in equilibrium with H_2_O_2_ (Figure 1), are also transformed to O_2_. The third approach using HOCl, by contrast, is particularly promising for the mechanistic evaluation of ozonation reactions in the laboratory because H_2_O_2_ is quantitatively transformed to O_2_ in a fast reaction with HOCl (*k* = 4.4. × 10^7^ M^−1^ s^−1^). O_2_ can subsequently be quantified by detecting the phosphorescence of ^1^O_2_ at 1270 nm.^5^ This method can also be applied to distinguish H_2_O_2_ from organic peroxides.

The goal of this study was to explore the utility of stable isotope-based approaches for the elucidation of the mechanisms of H_2_O_2_ formation during ozonation of olefinic and phenolic moieties. To this end, the two main objectives were (1) the development and implementation of an analytical procedure for the quantification of ^18^O/^16^O ratios in H_2_O_2_ through its conversion to O_2_ with the ensuing O isotope ratio measurements by established methods^33−35^ and (2) the assessment of O isotope fractionation of H_2_O_2_ formed through well-defined ozone reactions via Criegee intermediates to H_2_O_2_ for cinnamic acid^14^ and sorbic acid and more complex reactions pertinent to the formation of H_2_O_2_ from the ozonation of phenols^5,36,37^ and acrylic acid.^6^

## Materials and Methods

### Chemicals

All information related to reagents and solutions is provided in Section S1 in the Supporting Information.

### Experimental Conditions of Ozonation Reactions

#### Generation of Ozone Stock Solutions

Ozone (O_3_) stock solutions (1.6–1.9 mM) were obtained by a previously published procedure (Section S1).^10^

#### Ozonation of Model Compounds

Model compound solutions (phenol/phenolate, sorbic acid/sorbate, acrylic acid/acrylate, and cinnamic acid/cinnamate) were ozonated at pH 3 and 7 (10 mM phosphate buffer) in 100 mL serum bottles in the presence of DMSO (1–40 mM) with molar model compound-to-ozone ratios in the range of 3–5 (for concentrations see Table S1). DMSO was added as a hydroxyl radical (^•^OH) scavenger, to suppress ^•^OH reactions, which enables one to study the reactions of model compounds with ozone selectively. DMSO was selected because it has much lower yields of H_2_O_2_ from the reaction with ^•^OH compared to the typically used *tert*-butanol. During ozonation, *tert*-butanol yields up to 30% H_2_O_2_^8,38^ while H_2_O_2_ yields from DMSO are below 1%.^39^ The required DMSO concentration was estimated by calculating the scavenging efficiency (>95%), taking into account the apparent second-order rate constants for the reactions of model compounds and DMSO with ozone and ^•^OH at pH 3 and 7, respectively (Table S1).^10^ Experiments at pH 3 and 7 allowed studying of the pH dependences of product formation.

### Quantification of H_2_O_2_

The H_2_O_2_ concentrations of stock solutions were determined spectrophotometrically at 240 nm (ε = 40 M^–1^ cm^–1^)^40^ and in samples by the Allen's reagent method and via singlet oxygen (^1^O_2_) phosphorescence measurements depending on the selected model compound (see below).

In the Allen's reagent method, peroxides are quantified by a molybdate-catalyzed reaction with iodide to yield I_3_^–^ (351 nm, ε = 25700 M^–1^ cm^–1^).^41^ Based on iodide oxidation kinetics, this method can distinguish between different species (i.e., H_2_O_2_ and performic acid, measured after 1 min) and slower-reacting organic peroxides (measured after 20 min).^8,42,43^ The samples were collected in disposable semimicrocuvettes (PMMA, Brand, Germany) and measured on a UV spectrophotometer (Cary 100, Varian, USA) prior to and after 1 min and after 20 min of initiating the reaction. The LOD and LOQ are 0.4 and 1.1 μM H_2_O_2_, respectively. This method was only applied if organic peroxides were expected to be formed. In all other experiments, the ^1^O_2_ phosphorescence method was applied, in which H_2_O_2_ is quantified by ^1^O_2_ measurement (1270 nm, near-infrared photomultiplier tube (NIR-PMT)) produced during the reaction of HO_2_^–^ with HOCl.^5,7,30^ The detailed procedure for this method is provided in Section S2. For reproducible results, the equilibrium between organic peroxides vs H_2_O_2_ and the corresponding aldehydes must not be disturbed from the withdrawal of H_2_O_2_ during the transformation. Results from experiments with acrylic acid confirmed that the ^1^O_2_ method can successfully quantify H_2_O_2_ in the presence of organic peroxides (Table S3).

### Method for the Quantification of ^18^O/^16^O Ratios in H_2_O_2_

Oxygen isotope signatures of H_2_O_2_ (δ^18^O) were determined after its conversion to molecular O_2_ using the procedure outlined in Figure S1. The ^18^O/^16^O ratio of the resulting O_2_ was subsequently measured by gas chromatography isotope ratio mass spectrometry (GC/IRMS system consisting of a GC coupled via a Conflo IV interface to a Delta V Plus isotope ratio mass spectrometer) according to established procedures.^33−35^ Aqueous samples were treated in three principal, consecutive steps which included (a) the removal of residual dissolved O_2_ from the aqueous solution, (b) the conversion of H_2_O_2_ to O_2_, and (c) the transfer of gaseous samples into the GC/IRMS system with subsequent isotope ratio measurements. Details of the H_2_O_2_-to-O_2_-conversion procedure, its validation, and the consequences for accurate and sensitive determination of ^18^O/^16^O in H_2_O_2_ are described in Sections S3 and S4.

Briefly, after completion of ozonation experiments, the pH of the H_2_O_2_-containing solution in 100 mL serum bottles was adjusted to pH 3 with H_3_PO_4_ for stabilization and the sample purged with N_2_ (99.999%) for 10–15 min. The oxygen-free solutions were then redistributed into 20 mL crimp vials in an anoxic glovebox (O_2_ <0.1 ppm, UNIlab 2000, MBraun), leaving a maximum headspace of 400 μL. The headspace was used for addition of 50–200 μL of HOCl (1.5–1.7 M) once the reactors were removed from the glovebox and the injection of the same volume of ascorbic acid (2 M) immediately after HOCl addition, to quench residual HOCl. If the pH of the reacted solutions was <7, 9–20 μL of 5 M NaOH was added to adjust the pH to 7.0 for the conversion of H_2_O_2_ (Section S4.2). After conversion of H_2_O_2_ to O_2_, the extraction of O_2_ into the 3 mL N_2_-containing headspace was achieved by shaking the vials for 30 min at 200 rpm on an orbital shaker.^33−35^ δ^18^O values were obtained from ^18^O/^16^O ratio measurements of O_2_. As is detailed in Section S4, this value corresponds to the δ^18^O value of H_2_O_2_ due to complete H_2_O_2_-to-O_2_ conversion for H_2_O_2_ concentrations ≥3 μM (Figure S8) and ≥12 μM (Figure S9), depending on the absence and presence of DMSO and phosphate buffer, respectively (Section S4).

Evaluation of ^18^O/^16^O ratio measurements of O_2_ followed peak integration and blank correction procedures as described in detail previously^33,34^ and in Section S3.4.

Several factors such as time (i.e., for purging, on the stability of the involved species), pH, H_2_O_2_ disproportionation, side reactions, or purging have the potential to influence the H_2_O_2_ and O_2_ concentrations and δ^18^O values. No major influence was expected from these factors, which are discussed in detail in Section S4.

### Determination of δ^18^O Value of O_3_

δ^18^O of O_3_ was determined indirectly in a mass-balance approach through measurements of O isotope ratios of O_2_ by GC/IRMS. Given that O_3_ typically coexists with residual O_2_ in aqueous solutions, δ^18^O values of O_3_ (δ^18^O_O_3__) were derived from the comparison of δ^18^O from solution type (i) containing both O_3_ and O_2_ (δ^18^O_O_3_+O_2__) with δ^18^O of solution type (ii) where O_3_ was removed and only the residual O_2_ (δ^18^O_O_2__) remained.

Solutions of type (i) were O_3_ stock solutions in which O_3_ was converted into O_2_ by inducing an O_3_ decay chain reaction at pH 12 (eqs S1–S6), and the total O_2_ content was processed as described above and in Section S3. In solutions of type (ii), O_3_ was removed from stock solutions through the reaction of O_3_ with cinnamic acid. The remaining O_2_ was analyzed for ^18^O/^16^O ratios. The δ^18^O value of O_3_ was obtained in a mass balance calculation from eq 1 (see eqs S7–S12 for details).
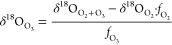
1

Note that the estimate for δ^18^O of O_3_ relies on the accurate quantification of O_3_ and O_2_ concentrations which are needed to calculate the fractional concentration (*f*_O_3__ and *f*_O_2__). O_3_ concentrations were determined as described in Section S1, and O_2_ concentrations in the O_3_ stock solutions were derived through estimates of O_3_ and O_2_ partial pressures in the ozone-containing oxygen gas as detailed in Section S5 (eqs S7–S12).

### Quantification of Model Compounds and Byproducts

Concentrations of phenol, cinnamic acid, benzaldehyde, and sorbic acid were measured by high-performance liquid chromatography coupled to a diode array detector (HPLC-DAD, Ultimate 3000, Thermo Scientific, Switzerland). Concentrations of acrylic acid were measured by ion chromatography (Dionex Integrion) with an IonPac AS19-4 μm column with an ^–^OH gradient and conductivity detection. Instrumental details, measurement ranges, and dilution factors are summarized in Table S2.

## Results and Discussion

### Formation of H_2_O_2_ and Organic Peroxides from the Reactions of Ozone with Olefins and Phenol

The yields of H_2_O_2_ and organic peroxides were determined using the two methods described in the section on quantification of H_2_O_2_, and the results are discussed below before discussing the resulting O isotopic signatures.

The yields of H_2_O_2_ and organic peroxides (as % O_3_ consumed) at pH 3 and 7 of the four selected model compounds vary significantly (Figure 3a and Table S3). The H_2_O_2_ yields for cinnamic acid (90 ± 5%, Figure 3a and Table S3) were similar to those in a previous study.^14^ For sorbic acid a H_2_O_2_ yield of close to 100% was also observed in this study. At pH 7, the H_2_O_2_ yields when using the ozonation of acrylic acid, a compound known to form organic peroxides, were comparable for the ^1^O_2_ method and the Allen's method with 52 ± 4% and 40.11 ± 0.01%, respectively (% of consumed O_3_). Slight differences in the yields might come from differences in time elapsed between the reactions and the H_2_O_2_ measurement, because H_2_O_2_ is in equilibrium with an organic peroxide. In a previous study, 58% H_2_O_2_ (pH 7) was reported for this reaction system^6^ and thus the value from ^1^O_2_ measurement is consistent. Notably, the total peroxide yield (H_2_O_2_ and organic peroxides) determined by the Allen's method was 78–80% for acrylic acid (Table S3). This can be explained by the slow reaction of hydroxymethylhydroperoxide in the Allen's method with incomplete reaction even after 20 min.^6,10,13^ Overall, H_2_O_2_ concentrations can be reliably determined for ozonated model compounds by the ^1^O_2_ method, and therefore this was applied for phenol, because the Allen's method cannot be applied due to interferences of phenol transformation products.^5^ The H_2_O_2_ yield (per mole of consumed O_3_) of phenols was on average 17 ± 1% at pH 7 and increased to 33 ± 2% at pH 3 (Figure 3c). These yields are comparable to those in previous studies (13–18% at pH 7 and 36% at pH 3).^5,36,37^ H_2_O_2_ yields from the reaction of ozone with phenol for pH 3–4.5 and 8 are provided in Table S4. Residual model compound concentrations upon ozonation are shown in Figures S10–S13. The molar consumption of model compounds per mole of O_3_ is between 1.09 and 0.94 for sorbic acid/sorbate and acrylic acid/acrylate, respectively (Figures S11 and S12), which is expected based on the Criegee mechanism. For phenol/phenolate, the range is between 0.49 and 0.53 (Figure S13), close to reported values.^5,37^

### Validation of the Experimental Procedure for δ^18^O Determination in H_2_O_2_

The reproducibility, accuracy, and precision of the experimental procedure for quantification of δ^18^O values in H_2_O_2_ were examined in three steps. First, the quantitative conversion of H_2_O_2_ to O_2_ was tested for the typical range of H_2_O_2_ concentrations in the experiments (≤120 μM). Second, the linear range and method detection limits (MDLs) for ^18^O/^16^O ratio measurements in O_2_ from the oxidation of H_2_O_2_ with HOCl were identified for experimental conditions representing typical concentrations used during olefin ozonation necessary to maintain a molar olefin excess relative to O_3_ and allow sufficient scavenging by DMSO (Table S1, Section S2). Finally, the procedure was validated by quantifying δ^18^O values of H_2_O_2_ from the well-characterized ozonation of cinnamic acid to benzaldehyde, glyoxylate, and H_2_O_2_.

Figure S2b shows that the conversion of H_2_O_2_ to O_2_ through addition of HOCl was close to stoichiometric with O_2_ yields of 90 ± 10% (Figure S2c) for H_2_O_2_ concentrations between 0 and 120 μM in ultrapurified water. Blank concentrations of dissolved oxygen were typically below 3 μM (Figure S2a) and were accounted for in background subtraction procedures in a stable O isotope analysis (Section S3.4). The efficient transformation to O_2_ led to an MDL for δ^18^O values of H_2_O_2_ in aqueous solution of 3 μM (Figure S8). Identical δ^18^O values were determined for the H_2_O_2_ concentration range up to 120 μM (Figure S8). The presence of 10 mM phosphate buffer and 5 mM of DMSO resulted in larger variations of ^18^O/^16^O ratio measurements of O_2_ and in a slightly elevated MDL of 12 μM (Figure S9). This MDL is consistent with those determined previously for δ^18^O of O_2_ in smaller sample volumes (10 mL vs 20 mL).^33,34^

Average δ^18^O values in H_2_O_2_ standards amounted to 21.9 ± 0.7‰ (*n* = 17, Figure S8b) in ultrapurified water. In a typical sample matrix, the δ^18^O values of these H_2_O_2_ standards were 22.2 ± 1.0‰ (*n* = 20) and thus identical within uncertainty (Figure S9b). All measured values coincide with the range of measured δ^18^O of H_2_O_2_ standards examined previously of 21.4–25.8‰.^27,44^ These O isotope signatures are confined to an amazingly narrow range of approximately 5‰, presumably because commercially available H_2_O_2_ is almost exclusively produced by the anthraquinone process.^45^ The agreement of the measurement with previous data for δ^18^O of H_2_O_2_ further underscores the accuracy of the presented analytical procedure.

**Figure 2:**
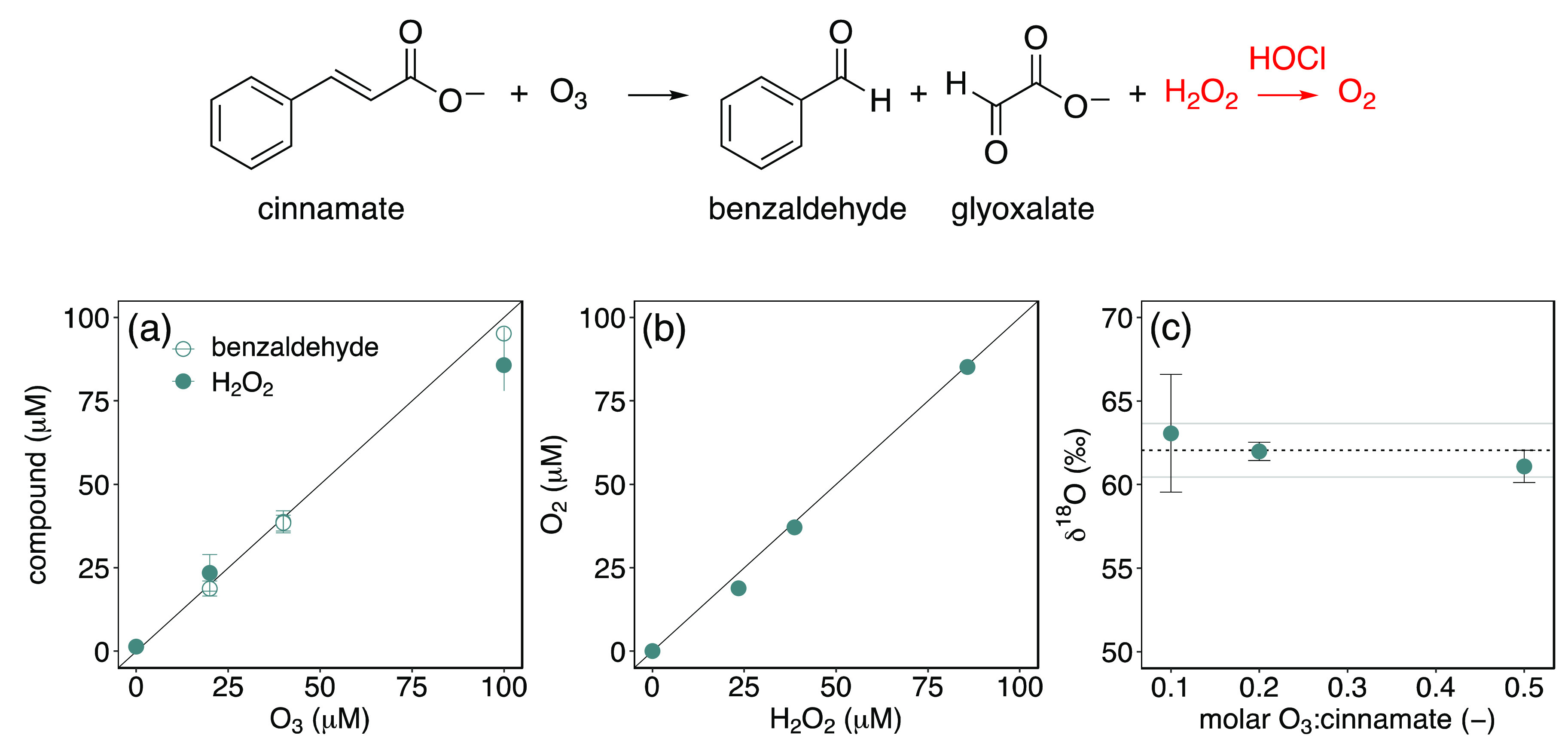
(top) Reaction of cinnamate with O_3_ leads to benzaldehyde, glyoxylate and H_2_O_2_ which can be transformed to O_2_ with the described chlorine-based procedure (indicated in red). (a) Formation of benzaldehyde (slope of 0.92 ± 0.02, *R*^2^ = 0.996, empty circles) and H_2_O_2_ (slope of 0.87 ± 0.03, *R*^2^ = 0.997, filled circles) as a function of increasing ozone doses. (b) Relationship between O_2_ formation and H_2_O_2_ (*R*^2^ = 0.997, transformed by HOCl). (c) Corresponding δ^18^O values as a function of increasing molar O_3_:cinnamate ratios. Lines in (a) and (b) indicate a 1:1 formation. The horizontal line in (c) indicates an average value of 61.3 ± 1.9%. Experimental conditions: 200 μM cinnamic acid, 10 mM phosphate buffer at pH 7, 5 mM DMSO, and O_3_ concentrations of 0, 20, 40, and 100 μM. Error bars indicate duplicate and triplicate measurements in (a) and (c), respectively.

The analytical procedure to determine O isotopes of H_2_O_2_ was applied to the reaction of ozone with cinnamate (Figure 2). Cinnamate was ozonated at three ozone doses (20, 40, and 100 μM), with an excess of olefinic compound to achieve stoichiometric H_2_O_2_ formation. These conditions corresponded to molar O_3_:olefin ratios of 0.1–0.5. Per mole of consumed cinnamate, 0.87 ± 0.03 mol of H_2_O_2_ and 0.92 ± 0.02 mol of benzaldehyde were obtained, in agreement with a previous study (Figure 2a).^14^ A correlation of applied ozone doses with cinnamate, benzaldehyde, and H_2_O_2_ formation is shown in Figure 2a. A similar correlation was obtained for measured H_2_O_2_ and O_2_ concentrations after addition of HOCl to the samples from cinnamate ozonation (Figure 2b).

The average δ^18^O values of H_2_O_2_ from ozonation of cinnamate was 61.3 ± 1.9‰ (Figure 2c). The three δ^18^O values of H_2_O_2_ are identical within measurement uncertainties. The large standard deviation of the δ^18^O value from experiments at low molar O_3_:cinnamate ratios was attributed to O_2_ concentrations approaching the MDL. Overall, the δ^18^O value is substantially higher than that of O_3_ (5 ± 1‰, Section S5) indicating an enrichment of ^18^O in H_2_O_2_. This phenomenon will be discussed in detail below.

Based on the validation of the analytical procedure with H_2_O_2_ standard solutions (Figures S8 and S9), it was concluded that δ^18^O can be determined reliably in experiments for the reaction of cinnamate with ozone (Figure 2c). The same analytical approach was applied to the model compounds sorbic acid, acrylic acid, and phenol (see below).

### Oxygen Isotopic Signatures of H_2_O_2_ Formed from Reactions of Ozone with Olefins and Phenol

**Figure 3:**
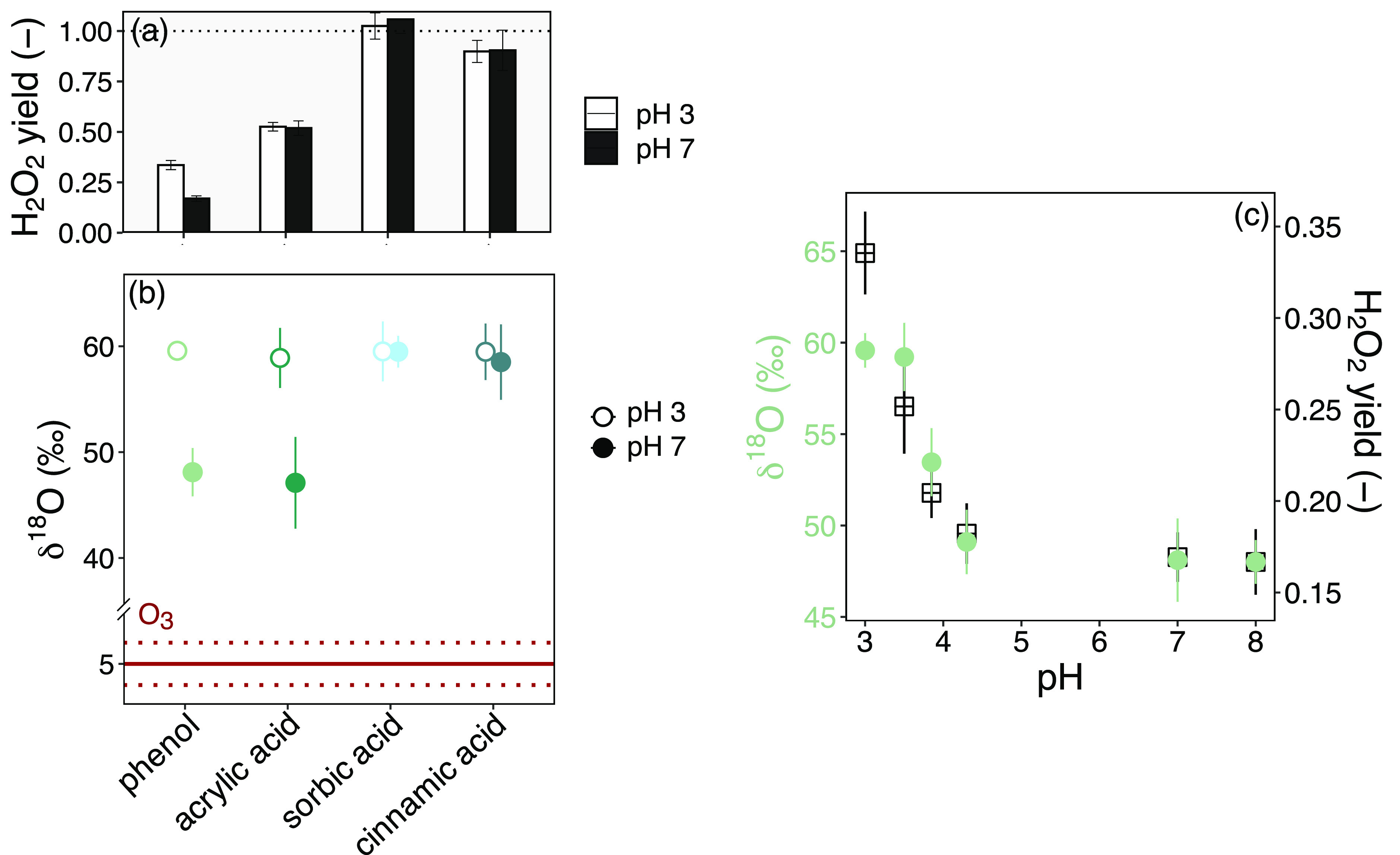
Reactions of ozone with phenol and olefinic model compounds. (a) H_2_O_2_ yields and (b) oxygen isotopic signatures of H_2_O_2_ formed from the reactions of ozone with phenol and olefinic model compounds at pH 3 (empty symbols) and 7 (filled symbols) and of O_3_ (dark red solid and dotted lines (standard deviation)). Please note the split axis between 5‰ and 40‰. (c) δ^18^O of H_2_O_2_ from ozonation of phenol (filled circles) and H_2_O_2_ yields (squares) for ozonation experiments at pH 3, 3.5, 3.85, 4.3, 7, and 8 (10 mM phosphate buffer). The pH is adjusted to pH 3 after ozonation to preserve H_2_O_2_ and adjusted to pH 7 for reproducible H_2_O_2_ to O_2_ turnover by HOCl. The number of replicates in all cases was ≥2 (see Table S5). Detailed information about ozonation of each compound is provided in Section S4.5.

The H_2_O_2_ yields (Figure 3a) and δ^18^O values of H_2_O_2_ formed in ozonation reactions of three olefins, acrylic acid, sorbic acid, and cinnamic acid, as well as phenol were evaluated at pH 3 and 7. A substantial O isotope fractionation between ozone (5 ± 1‰) and H_2_O_2_ was observed in all experiments, with ^18^O preferentially accumulating in H_2_O_2_. Figure 3b shows that the ozonation of all compounds at pH 3 (empty symbols) and of two olefins at pH 7 (filled symbols) resulted in identical O isotopic signatures of approximately 59‰ (average δ^18^O of 58.6 ± 2.6‰). For sorbic acid and cinnamic acid, which exhibited an H_2_O_2_ yield close to 100% (Figure 3a), the δ^18^O values were pH-independent. By contrast, for ozonation of phenol and acrylic acid an identical δ^18^O value of H_2_O_2_ was only observed at pH 3.0.

At pH 7, the δ^18^O values of H_2_O_2_ from phenol and acrylic acid ozonation were 48.8 ± 2.8‰ and 47.1 ± 4.3‰, respectively. The δ^18^O value of H_2_O_2_ from the ozonation experiments with phenol was also evaluated at intermediate pH values as shown in Figure 3c. Between pH 3.5 and 4.3, δ^18^O of H_2_O_2_ gradually decreased from approximately 59‰ to 49‰ before reaching a constant value up to pH 8.0. For pH > 3.5, δ^18^O of H_2_O_2_ correlated with the moderate decrease of H_2_O_2_ yield from 0.25 to 0.20 (Table S4). Only at pH 3.0 did this correlation of δ^18^O of H_2_O_2_ with its yield become invalid.

### H_2_O_2_ Formation from Cinnamate and Sorbate: Baseline Case

**Figure 4:**
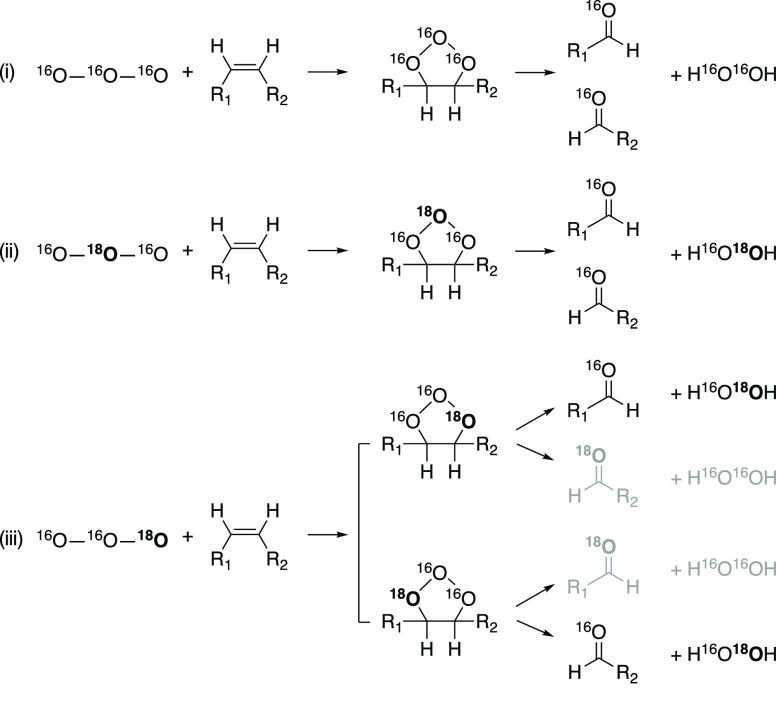
Ozonation of olefins by the Criegee mechanism. Isotopologues and isotopomers of O_3_, the Criegee ozonide and the ensuing formation of carbonyl compound and H_2_O_2_. Preferential bond cleavage of ^16^O–^16^O bonds in the Criegee ozonide leads to an enrichment of ^18^O in H_2_O_2_ compared to O_3_. Due to their low abundance, multiply substituted heavy O_3_ was not taken into account. Cases (i–iii) designate the reactions of the different isotopologues and isotopomers. Black products are preferentially formed compared to gray products.

During ozonation of the olefins cinnamic and sorbic acid (in molar excess to ozone), O_3_ is transformed stoichiometrically to H_2_O_2_ and the corresponding carbonyl compounds (Figures 1 and 2). Therefore, the isotopically heavier H_2_O_2_ (∼59‰) compared to O_3_ (5 ± 1‰) (Figure 3b) has to result from the abundance of the different ^16^O- and ^18^O-containing species and the reactions with which heavy and light O atoms are transferred to H_2_O_2_. Figure 4 illustrates this phenomenon conceptually by considering that O_3_ not only consists of ^16^O and ^18^O (isotopologues ^16^O_3_ vs ^16^O_2_^18^O) but also that the ^18^O isotopologue of O_3_ consists of two isotopomers where ^18^O can be located at the central or edge O atom (^16^O^18^O^16^O, ^16^O^16^O^18^O). In case (i), from the Criegee reaction of ^16^O_3_, only isotopically light H_2_O_2_ is formed. In case (ii), for ^16^O^18^O^16^O, all the ^18^O will be transferred to H_2_O_2_. In case (iii), for ^16^O^16^O^18^O the efficiency of the ^18^O transfer to H_2_O_2_ is determined by the frequency of cleaving bonds between ^16^O–^16^O relative to ^16^O–^18^O.

The observation of ^18^O-enriched H_2_O_2_ is consistent with the notion that bond dissociation energies are smaller for bonds containing light isotopes.^46^ The ozonide bond thus breaks preferentially between ^16^O–^16^O atoms (Figure 4, case (iii)), resulting in a larger share of ^18^O from the ^18^O-containing ozonide being transferred to H_2_O_2_ compared to O_3_, while a higher fraction of ^16^O is recovered in the formed carbonyl groups. Note that no further O–O bond cleavage occurs in the path to H_2_O_2_. This behavior of preferential reactions of bonds containing light isotopes corresponds to a normal kinetic isotope effect (KIE > 1) and suggests that the cleavage of the O–O bond in the ozonide is the source of O isotope fractionation. However, specific information about the magnitude of O–O bond cleavage isotope effects in ozonide intermediates and of the following reactions leading to H_2_O_2_ formation are not available. Here, this normal KIE was observed for the ozonation of all model compounds, but the extent of ^18^O fractionation between O_3_ and H_2_O_2_ was different for phenol and acrylic acid at pH 7 as compared to all other cases (Figure 3b). Based on these findings, it is hypothesized that the ozonation of acrylic acid and phenol deviates from the baseline case. In these cases, possibly reaction steps other than those of the Criegee mechanism lead to a smaller enrichment of ^18^O in H_2_O_2_.

### H_2_O_2_ Formation from Ozonation of Acrylic Acid

**Figure 5:**
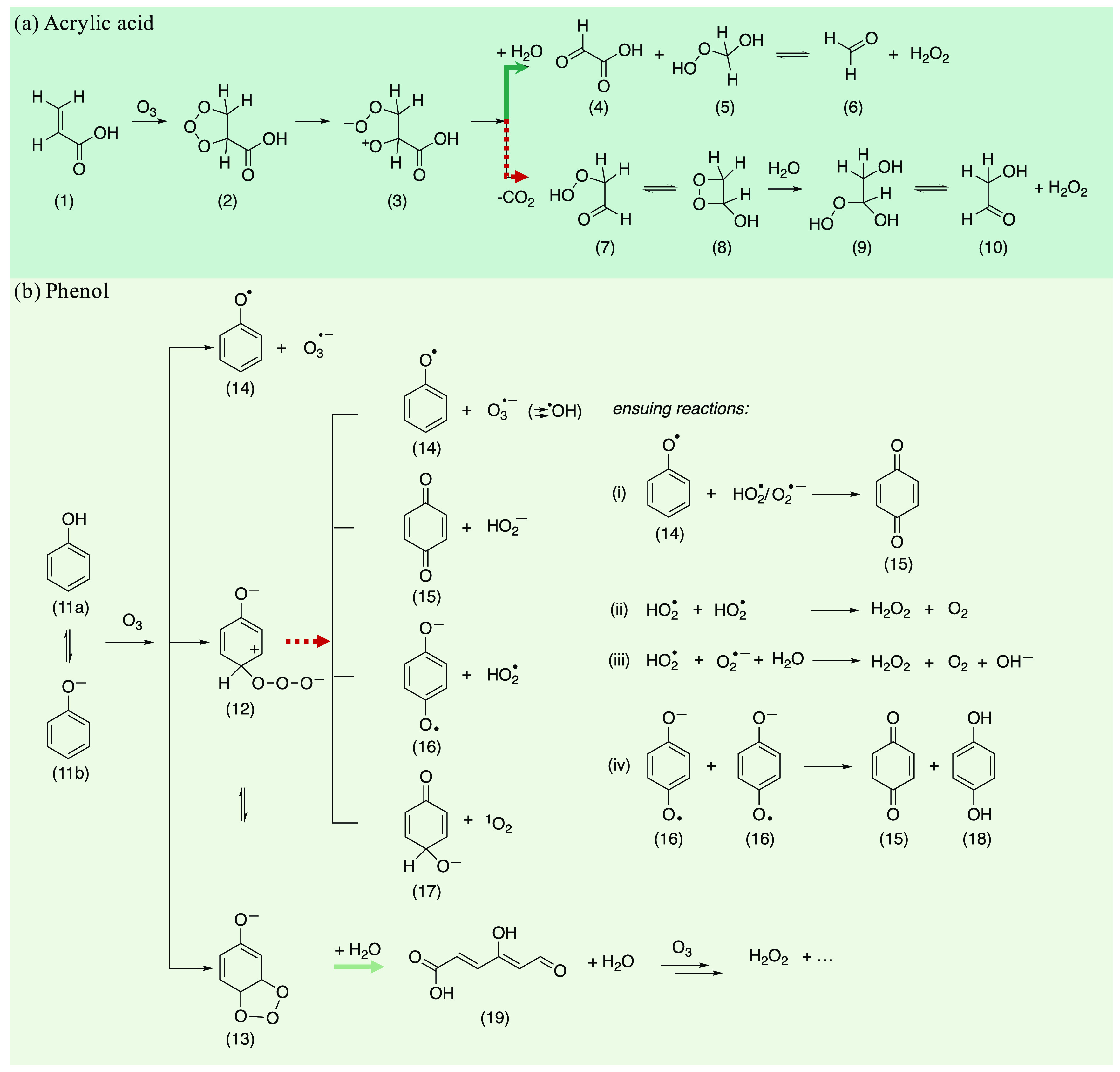
Mechanisms for the reactions of ozone with (a) acrylic acid and (b) phenol. Green arrows indicate Criegee-type pathways. Red dotted arrows indicate other pathways.

The ozonation of acrylic acid deviates from the baseline case in that the δ^18^O of H_2_O_2_ is less than 59‰ at high pH (Figure 3b) and the H_2_O_2_ yields are significantly less than 100% (Figure 3a). The reaction mechanism for the ozonation of acrylic acid is shown in Figure 5a with a pH-dependent branching (formation of products **4** and **7**).^6^ In the upper pathway, glyoxylic acid (**4**) is formed alongside hydroxymethylhydroperoxide (**5**), which is in equilibrium with formaldehyde (**6**) and H_2_O_2_. In the lower pathway (red dotted arrow) the Criegee-type zwitterion undergoes decarboxylation, leading to 2-hydroperoxyacetaldehyde (**7**) as the organic peroxide species. Glycolaldehyde (**10**) and H_2_O_2_ are then formed by hydrolysis of the dioxetane (**8**).^6^

The pH dependence of the two pathways was previously determined by measuring the formaldehyde yield (**6**) as a function of the pH.^6^ A formaldehyde fraction of 0.72 at pH 2 and 0.52 at pH 7 indicates that the decarboxylation pathway becomes more important at higher pH. However, the present study shows that the yields of H_2_O_2_ were similar at both pH values (52%, Figure 3a) and are consistent with previous H_2_O_2_ measurements for pH 7 (58% yield).^6^ The finding of greater ^18^O enrichment in H_2_O_2_ at lower pH (Figure 3b) implies that the decay of the Criegee ozonide cannot be solely responsible for the observed ^18^O enrichment. Both mechanisms proceed through the same Criegee ozonide and the same ensuing zwitterion. The main differences between the two mechanisms are the yields of the carbonyl-containing products formaldehyde (**6**) and glycolaldehyde (**10**). These compounds are in equilibrium with the corresponding organic peroxides **5** and **9**, which together with H_2_O_2_ make up 100% of the consumed ozone.^6^ Organic peroxides thus not only account for the 48% share of O_3_ atoms that did not wind up in H_2_O_2_ (Table S3) but could also determine the δ^18^O of H_2_O_2_ through an O isotope fractionation pertinent to the equilibrium between organic peroxides and aldehydes/H_2_O_2_ (**5** ⇄ **6** + H_2_O_2_, **9** ⇄ **10** + H_2_O_2_, Figure 5a). The correlation of lower δ^18^O of H_2_O_2_ with higher pH and an increased contribution of the decarboxylation pathway suggest that the smaller O isotope fractionation could arise from the equilibrium **9** ⇄ **10** + H_2_O_2_. The enthalpy of formation of RH_2_C–OOH bonds varies as a function of R.^8^ It increases from R = H to R = CH_3_ from −139 to −175.4 kJ/mol. Therefore, it can be expected that the C–OOH bond is stronger for glycolaldehyde (containing an ethyl group) than for formaldehyde (containing a methyl group), which would lead to a preferential bonding of the ^18^OOH and therefore a lower δ^18^O in the H_2_O_2_ in equilibrium at pH 7 compared to pH 3. It is interesting to note that the reaction **5** ⇄ **6** + H_2_O_2_ leads to an isotopic composition similar to that for H_2_O_2_ formed during the stoichiometric ozonation of cinnamate or sorbate, where no organic peroxides accumulate. At this point, there is not sufficient information to explain this observation.

### H_2_O_2_ Formation from Ozonation of Phenol

At pH 7, the H_2_O_2_ yield from ozonation of phenol is much lower at 17% (Figure 3a) and the δ^18^O of H_2_O_2_ (∼49‰) deviates significantly from the baseline case (59‰) at pH 3 (Figure 3b). In contrast to acrylic acid (similar δ^18^O of H_2_O_2_ at pH 7), where the mechanism at both pH values proceeds through the same Criegee ozonide and the ensuing zwitterion, phenol can react with ozone via a monodentate (**11** → **12**) or bidentate (**11** → **13**) attack or an outer sphere electron transfer (**11** → **14**) (Figure 5b). The formed ozone adduct **12** can react further via different pathways: release of an ozonide radical anion (O_3_^•–^) (**14**), hydrogen peroxide (p*K*_a_(H_2_O_2_) = 11.8^47^) (**15**), hydroperoxyl radical (p*K*_a_ (HO_2_^•−^) = 4.8^48^) (**16**), and singlet oxygen (^1^O_2_) (**17**).^5,7,10,36,37^

Apart from the Criegee-type mechanisms (reaction products from ozonation of **19**, Figure 5b), H_2_O_2_ can also be formed from **12** (Figure 5b) by a direct rearrangement with heterolytic bond cleavage of the O–O bond, leading to H_2_O_2_ and benzoquinone (**12** → **15**) or homolytic bond cleavage of the O–O bond (**12** → **16**) which leads to benzoquinone and/or H_2_O_2_ by various ensuing reactions ((i)–(iv) in Figure 5b).^5,36^ Overall, reactions with phenols (equilibrium of phenol (**11a**) and phenolate (**11b**), p*K*_a_ 9.9) offer not only multiple pathways to H_2_O_2_ in a sequence of pH-dependent reactions but also pathways which compete with H_2_O_2_ formation.

At pH 3, about 90% of the ozone reactions occur with phenol and only 10% with phenolate, whereas the fraction of the phenolate reactions increases dramatically with increasing pH (>99% at pH 7, Figure S15). Estimations of Gibbs free energies show that for neutral phenol, the bidentate addition of O_3_ (**11a** → **13**) is thermodynamically favored.^5^ For phenolate, the monodentate attack and a rearrangement of a bidentate form to the noncyclic form are both favored (**11b** → **12** and **12** → **13**, respectively).^5^ Consequently, there is a distinction of predominance of the pathways at different pH values potentially leading to differences in δ^18^O of H_2_O_2_.

At pH 3, 2 mole of ozone are consumed per mole of phenol (Figure S13). Consequently, further reactions with transformation products are expected, such as with muconic acid, which has an apparent second-order rate constant for the reaction with ozone that is one order of magnitude higher than for phenol at pH 3 (*k* = 1.3 × 10^4^ M^–1^ s^–1^ (muconic acid) vs *k* = 1.5 × 10^3^ M^–1^ s^–1^ (phenol)).^6,49^ Under these conditions the higher H_2_O_2_ yields (33%) compared to pH 7 (17%) is caused by H_2_O_2_ formation by a Criegee-type mechanism from muconic acid (**19**). Potential H_2_O_2_ formation with concomitant benzoquinone formation is only minor (15% benzoquinone yield at pH 3 in % of consumed O_3_^5^). Thus, it is posited that H_2_O_2_ formation at pH 3 is mainly based on Criegee-type reactions leading to a δ^18^O of H_2_O_2_ similar to that for the baseline case.

With increasing pH, the H_2_O_2_ yields and the determined δ^18^O of the formed H_2_O_2_ clearly decrease (Figure 3c) with an inflection point at around pH 3.85. At this pH, phenol and phenolate exhibit the same kinetic contribution to the oxidation of total phenol by ozone with the same apparent second-order rate constants (Figure 3c and Figure S15).^10^

At pH 7, H_2_O_2_ is mainly formed by concomitant benzoquinone formation (30% benzoquinone yield at pH 7 in % of consumed O_3_^5^), where higher benzoquinone yields compared to H_2_O_2_ may arise from ensuing reactions ((i) and (iv) in Figure 5b). The lower yields of H_2_O_2_ at pH 7 (17%) compared to pH 3 (33%) might be caused by competing ozone reactions without H_2_O_2_ formation, which may also influence the δ^18^O of H_2_O_2_. A case in point is the loss of ^1^O_2_ from the ozone adduct (**12** → **17**, Figure 5b). ^1^O_2_ yields at pH 7 are around 5–6%, while ^1^O_2_ was not detected at pH 3.^36^ In addition, pathway **12** → **14** is more pronounced at pH 7 than at pH 3, which can be concluded from the corresponding ^•^OH yields (pH 3 (∼20%), pH 7 (∼30%)).^36^

The transfer of oxygen atoms from O_3_ to H_2_O_2_ and other reactive oxygen species from the ozone adduct **12** substantially differs from the baseline case, which involves the Criegee ozonide (Figure 4). Figure S14 shows the fate of the different ozone adduct isotopologues and isotopomers. For the Criegee ozonide isotopologues, a preferential bond cleavage of ^16^O–^16^O leads to the transfer of all ^18^O atoms to H_2_O_2_ (Figure 4). During ozonation of phenolate, only three out of four ozone adduct isotopologues and isotopomers transfer ^18^O to H_2_O_2_ and other reactive oxygen species (Figure S14a–c). The ozone adduct isotopomer (Figure 14d) with a C–^18^O bond will lead to a loss of ^18^O to the oxygen-containing aromatic products. Consequently, less ^18^O is transferred to H_2_O_2_ and other reactive oxygen species compared to the baseline case, which can explain the lower δ^18^O of H_2_O_2_. Furthermore, competing pathways enhance this effect as ^18^O can be lost, which is then no longer available for H_2_O_2_ formation. For example, electron transfer (**12** → **14**) leads to a loss of ^18^O to O_3_^•–^ for all heavy isotopomers ((i), Figure S14a–d) and thus is not available for H_2_O_2_ formation, leading to an even lower δ^18^O of H_2_O_2_ compared to the baseline case.

Overall, comparing the different pathways that contribute to the H_2_O_2_ budget at pH 3 and 7, it can be concluded that Criegee-type reactions are more pronounced at pH 3 and mostly control the observed δ^18^O of H_2_O_2_ of ∼59‰. The agreement of this value with H_2_O_2_ from olefin ozonation might be fortuitous. At pH 7, H_2_O_2_ is mainly formed via benzoquinone formation. However, the competing ozone-consuming reactions electron transfer (**12** → **14**, which leads to ^•^OH formation) and the loss of ^1^O_2_ (**12** → **17**) lead to lower H_2_O_2_ yields. Overall, the lower δ^18^O of H_2_O_2_ of ∼49‰ at pH 7, compared to the baseline case (Figure 4), is governed by (1) a lower expected δ^18^O of H_2_O_2_ from the benzoquinone formation pathway (Figure S14), (2) formation of ^1^O_2_ (**12** → **17**), and (3) consumption of ozone without H_2_O_2_ formation and loss of ^18^O by the electron transfer process (**12** → **14**).

## Implications

A novel method for the measurement of the oxygen isotope composition of H_2_O_2_ has been developed. This method was applied to investigate the oxygen isotopic composition of H_2_O_2_ formed during ozonation of olefins and phenol. It was found that δ^18^O of H_2_O_2_ is significantly higher (>40‰) than in ozone for all precursors. Whereas for ozonation at pH 3 the δ^18^O of H_2_O_2_ was the same for all precursors, at pH 7, the δ^18^O of H_2_O_2_ was 10‰ lower for ozonation of acrylic acid and phenol. This observation opens a potential option for pH-dependent H_2_O_2_ precursor elucidation in more complex compound mixtures such as dissolved organic matter (DOM). It is expected that olefins with an acrylic acid type ozonation chemistry are rare in such matrices and that the ozone chemistry is mainly determined by phenols and olefins reacting by a standard Criegee mechanism. Under these conditions, the pH-dependent concentration and δ^18^O of H_2_O_2_ could potentially yield information on the respective precursors, which are also important for the formation of undesired carbonyl compounds.^12^ However, to use O isotope fractionation trends in this manner, a more extensive and more rigorous assessment of ozonation of various olefins and substituted phenols and mixtures thereof in terms of pH-dependent H_2_O_2_ yields and δ^18^O of H_2_O_2_ needs to be performed. Additionally, the ozonation of standard DOM samples and DOM from environmental water samples should be explored to assess the feasibility of the proposed approach. A similar conceptual approach has been successfully applied to elucidate precursors of chloroform formation during chlorination of model compounds and real water samples.^25^ Furthermore, there are many reactions in environmental (bio)chemistry where H_2_O_2_ is involved and the novel method for O isotope analysis reported here could be applied to gain more mechanistic insights into processes involving reactive oxygen species.^1,3,4,50^

## Supporting Information Available

Reagents, solutions, and instrumental details, overview of measurement of model compounds, analytical approach for the determination of δ^18^O in H_2_O_2_, application of isotopic H_2_O_2_ characterization to ozonation experiments, approach for the derivation of δ^18^O in O_3_, and background information to explain isotopic signatures (PDF)
