# Supplementary material for: Hydrogen Peroxide Formation during Ozonation of Olefins and Phenol: Mechanistic Insights from Oxygen Isotope Signatures
Source: Environ Sci Technol. 2023 May 8;57(47):18950–9. doi: 10.1021/acs.est.3c00788 (PMC10690717; doi:10.1021/acs.est.3c00788)
Supplement: Supplementary file 1 — es3c00788_si_001.pdf [file es3c00788_si_001.pdf]

Supplementary Information

Hydrogen peroxide formation during ozonation of olefins and phenol: Mechanistic insights from oxygen isotope signatures

Joanna Houska,[1,2] *Laura Stocco,*[1,2] *Thomas B. Hofstetter,*[1,3*] *Urs von Gunten*[1,2,3*]

[1]Eawag Swiss Federal Institute of Aquatic Science and Technology, 8600 Dübendorf, Switzerland

[2] School of Architecture, Civil, and Environmental Engineering, École Polytechnique Fédérale de Lausanne, 1015 Lausanne, Switzerland

[3] Department of Environmental System Science, ETH Zurich, 8092 Zurich, Switzerland

[*] Corresponding authors: vongunten@eawag.ch (U. von Gunten), thomas.hofstetter@eawag.ch (T. B. Hofstetter).

24 pages, 15 Figures, 5 Tables

Table of Contents

Section S1. Reagents, solutions and instrumental details *3*

Section S2. Overview of measurements of model compounds *4*

Section S3. Analytical approach for the determination of $\delta^{18}O$ in H_2O_2 .... *5*

S3.1 Preparation of samples for $^{18}O/^{16}O$ ratio determination in H_2O_2 .... **5**

S3.2 Dissolved oxygen measurements **7**

S3.3 Oxygen isotope ratio measurements by GC/IRMS **8**

S3.4 Data evaluation **9**

Section S4. Application of isotopic H_2O_2 characterisation to ozonation experiments *10*

S4.1 Conservation of H_2O_2 prior to conversion to O_2 .... **10**

S4.2 Effect of solutes on HOCl-based H_2O_2 conversion to O_2 .... **12**

S4.3 Effects of solution constituents on $^{18}O/^{16}O$ ratio measurements by GC/IRMS **13**

S4.4 Method detection limits for $^{18}O/^{16}O$ ratio measurements in H_2O_2 .... **14**

S4.5 Overview of model compound analysis and $^{18}O/^{16}O$ ratio measurements **16**

Section S5. Approach for the derivation of $\delta^{18}O$ in O_3 .... *19*

Section S6. Background information to explain isotopic signatures *23*

References *24*

Section S1. Reagents, solutions and instrumental details

Chemicals. Hydroxyl radical (•OH) scavenger dimethyl sulfoxide (DMSO, anhydrous, ≥ 99.9%), sodium hydroxide solution (≥98%), sodium phosphate dibasic dihydrate (≥98%), sodium phosphate monobasic monohydrate (99%), benzaldehyde (>99.5%), L-ascorbic acid (reagent grade), trans-cinnamic acid (≥ 99%), acrylic acid (anhydrous, 99%), methacrylic acid (99%), cobalt(III) nitrate hexahydrate (≥ 98%), sodium hypochlorite solution (6-14% active chlorine basis) and hydrogen peroxide solution (purum p.a., ≥35%) were obtained from Sigma-Aldrich (Switzerland). Potassium hydrogen phthalate (≥ 99.5%) and sorbic acid (99%) were obtained from Fluka, Switzerland. Ammonium heptamolybdate tetrahydrate (≥ 99%) was obtained from Merck. Phenol (99.5%) as well as methanol (99.99%) and acetonitrile (99.99%) both LC/MS grade were obtained from Thermo Fisher Scientific (Switzerland). All aqueous solutions were prepared with ultra-purified water with a resistivity of > 18.2 MΩ·cm, ASTM type 1) from arium® pro Ultrapure Laboratory Water Systems, Sartorius.

Model compound solutions. Model compounds were dissolved in ultrapurified water at concentrations between 40-60 mM. For acrylic acid, sorbic acid and cinnamic acid, an equivalent molarity of NaOH was added, to allow dissolution. The solutions were prepared freshly every 4 weeks.

Generation of ozone stock solutions. Ozone (O_3) stock solutions (1.6-1.9 mM) were obtained by producing ozone-containing oxygen gas by an ozone generator (BMT 803 BT, BMT Messtechnik, Berlin) from pure oxygen (Carbagas, 99.995%) which was bubbled into ice-cooled ultra-purified water.[1] The ozone concentration was determined spectrophotometrically at 260 nm ($\varepsilon = 3200\ M^{-1}cm^{-1}$). [2]

Section S2. Overview of measurements of model compounds

Table S1. Model compound and DMSO concentrations used in the experiments based on the apparent second-order rate constants *k* for the reactions with $^{\bullet}OH$ and O_3. Indication of pH and applied O_3 dose (c_{O3}).

Model compound	C (µM)	c_{O3} (µM)	pH	$k_{OH, app}$ ($M^{-1}s^{-1}$)	$k_{O3, app}$ ($M^{-1}s^{-1}$)[a]	DMSO (mM)[b]
phenol	600	~267	3	$1.4 \cdot 10^{10}$	$1.5 \cdot 10^{3}$	25
phenol	800	~267	7	$1.4 \cdot 10^{10}$	$1.8 \cdot 10^{6}$	33
acrylic acid	600	~120	3	*$8.4 \cdot 10^{9}$*	$3.5 \cdot 10^{4}$	15
acrylic acid	600	~120	7	*$8.4 \cdot 10^{9}$*	$1.6 \cdot 10^{5}$	15
sorbic acid	200	~45	3	*$8.1 \cdot 10^{9}$*	$3.3 \cdot 10^{5}$	5
sorbic acid	200	~45	7	*$8.1 \cdot 10^{9}$*	$9.6 \cdot 10^{5}$	5
cinnamic acid	200	~45	3	$8.1 \cdot 10^{9}$	$6.2 \cdot 10^{4}$	5
cinnamic acid	200	~45	7	$8.1 \cdot 10^{9}$	$3.8 \cdot 10^{5}$	5

[a] the apparent second-order rate constants for the reactions with O_3 were calculated based on the known species-specific second-order rate constants and the respective pK_a.[2] [b] DMSO reactivity with $^{\bullet}OH$ and O_3 is $7 \cdot 10^9$ and 8 $M^{-1}s^{-1}$, respectively [3,4] and its concentration was determined to obtain a $^{\bullet}OH$ scavenging efficiency of > 95%. Italic values represent an estimate.

Table S2. Measurement details for the model compounds.

Model compound	Dilution factor for analysis	Analytical system	Eluents (%/%)	Flow rate (mL/min)	Detection λ (nm)
phenol	60	HPLC/DAD	Gradient (40-95% 10 mM H_3PO_4 / 5-60% acetonitrile	0.8	273
acrylic acid	200	IC-CD	Gradient (10-100mM KOH)	0.25	NA
sorbic acid	5	HPLC/DAD	15% acetonitrile/85% 30 mM acetate	0.6	240
benzaldehyde[a]	5	HPLC/DAD	40% methanol/60% 10 mM H_3PO_4	0.8	250

[a] benzaldehyde was measured instead of cinnamic acid.

Measurement procedure of H_2O_2 quantification by the singlet oxygen method. The procedure for the quantification of H_2O_2 by the singlet oxygen method proceeded as follows: (1) addition of 1 mL sample to a 1cm quartz cuvette and closing with a lid containing a syringe hole, (2) addition of 1 mL 50 mM chlorine in 100 mM phosphate buffer at pH 7.0 to a syringe, (3) start measurement of the counting unit (gate time 5 ms, 2000 measurement points, C8855-01, Hamamatsu Photonics K.K Electron Tube Division), (4) fast injection of the chlorine

solution by the syringe to the quartz cuvette,[5] (5) recording and integrating peaks with the spline method and processed in R (LOQ = 2.5 µM).

Table S3. Ozonation of model compounds: H_2O_2 yields at pH 3 and 7 determined by the singlet oxygen method.

Model compound (MC)	H_2O_2 (% of consumed O_3)		Organic peroxides (% of O_3 consumed)		Literature values (H_2O_2/ organic peroxides)
	pH 3	pH 7	pH 3	pH 7	
Cinnamic acid	90 ± 5	90 ± 1	NA	NA	100/NA (pH 6.5)[6]
Sorbic acid	102 ± 6	106 ± 7	NA	NA	NA
Acrylic acid	53 ± 2<br>37.90 ± 0.02[a]	52 ± 4<br>40.11 ± 0.01[a]	38.2 ± 0.1[a]	41.7 ± 0.4[a]	58 / 43 (pH 7)[7]
phenol	33 ± 2	17 ± 1	NA	NA	36[b]/NA (pH 3), 18[b]/- (pH 7)[8]<br>13/NA (pH 6-7)[9]

[a] by Allens reagent method, [b] in the presence of *t*-butanol as scavenger

Table S4. H_2O_2 yields (% of consumed O_3) for the reaction of phenol with ozone at pH values between 3 and 8.

	pH 3	pH 3.5	pH 3.85	pH 4.3	pH 7	pH 8
Phenol<br>this study	33 ± 2	25 ± 3	20 ± 1	18 ± 2	17 ± 1	17 ± 2
Phenol literature	36 [8]				18 [8]	

Section S3. Analytical approach for the determination of $\delta^{18}O$ in H_2O_2

S3.1 Preparation of samples for $^{18}O/^{16}O$ ratio determination in H_2O_2

The analytical procedure for the determination of $\delta^{18}O$ values of H_2O_2 is described in Figure S1. Steps 1-4 serve to transform H_2O_2 to gaseous O_2 while minimizing the contamination by

ambient O_2. Steps 5-6 involve a previously established methodology for measurement of $^{18}O/^{16}O$ ratios in O_2 by gas chromatography isotope ratio mass spectrometry (GC/IRMS).[10,11]

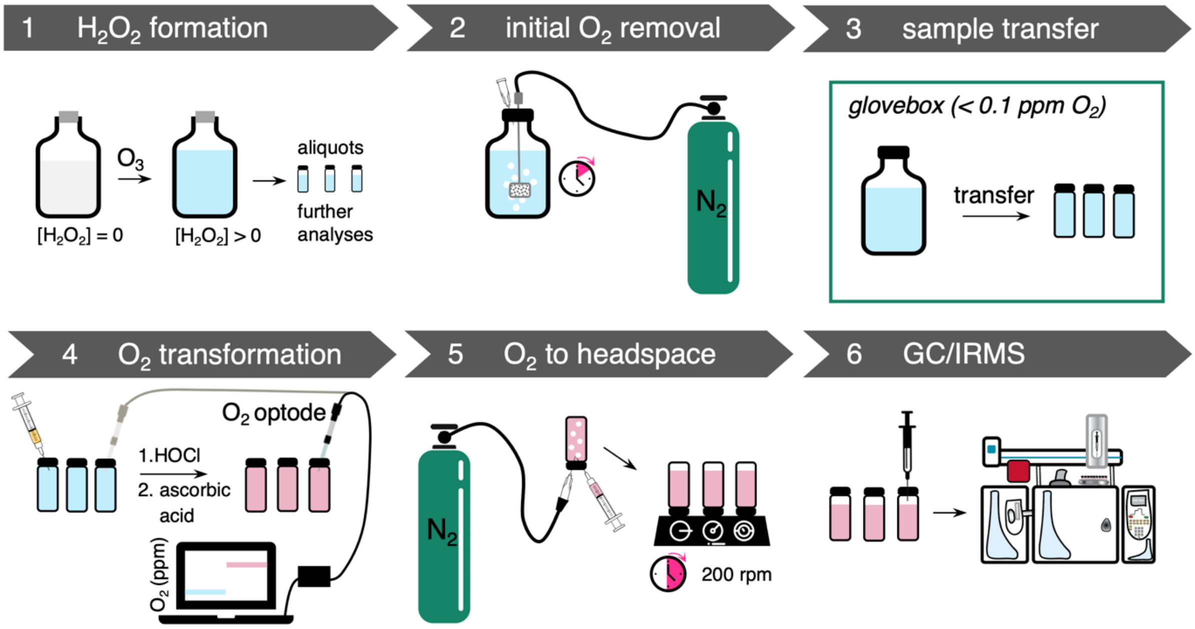

Figure S1. Schematic representation of the experimental procedure to transform H_2O_2 to O_2 for oxygen isotope ratio analyses.

The six procedural steps are as follows.

1. A standard solution contained up to 120 µM of H_2O_2 in 100 mL aqueous solution of 10 mM phosphate buffer in 100 mL serum bottles. Samples from ozonation experiments of different target compounds were prepared in 10 mM phosphate buffer and acidified instantly at the end of an experiment with phosphoric acid. From these reactors, aliquots of up to 10 mL were removed to determine the H_2O_2 concentration and measure residual concentrations of olefins/phenols and selected organic products.

2. The H_2O_2-containing samples (in serum bottles) were purged for 10-15 min with N_2 (sufficient time for 90-110 mL of solution) using a silicone frit inserted in a butyl stopper, to remove dissolved O_2.

3. The 100 mL serum bottles were closed with butyl stoppers, crimped and transferred to 21 mL headspace vials in an anaerobic glovebox under N_2 atmosphere ($O_2 < 0.1$ ppm, UNIlab 2000, M.Braun, Germany), leaving a maximum headspace of 400 µL.

4. The transformation of H_2O_2 to O_2 by HOCl was performed by maintaining the solution pH at 7 (pH was monitored before and after transformation for each experiment). Typically, 50-200 µL of HOCl solution (~1.5-1.7 M) and the same volume of 2 M ascorbic acid were used and injected sequentially using gas-tight Hamilton syringes. Both HOCl and ascorbic acid solutions were prepared freshly for each experiment in 12 mL headspace vials while purging of the headspace with N_2. Additionally, aliquots of 5 M NaOH were injected to adjust the pH to pH 7 (NaOH was added to the same syringe as HOCl and thus injected just prior to HOCl).
5. A headspace was created by manually replacing 3 mL of the sample solution with N_2 while holding the vials upside down.[12] Hereby, a small overpressure of 1.3-1.4 bar was created to minimize transfer of atmospheric O_2 into the vials. After creation of headspace, the vials (still in upside down position) were placed on an orbital shaker for 30 min at 200 rpm to facilitate transfer of O_2 to the gas phase.
6. Immediately after the O_2 extraction into the headspace, the samples were placed on the PAL-autosampler of the GC/IRMS device and 1500 µL of each headspace was injected. Single injections of different 21 mL vials served as replicates. To account for O_2-contamination, control samples (blanks in same sample matrix containing phosphate buffer, HOCl and ascorbic acid) were prepared in the same manner, but omitting H_2O_2 addition or generation through ozonation. Details regarding the instrumental analysis are described in Section S3.3.

S3.2 Dissolved oxygen measurements

Dissolved O_2 concentrations were measured before and after transformation of H_2O_2 to O_2 in one of the replicates, using a needle-type oxygen microsensor (NTH-PSt7, PreSens Precision Sensing GmbH, Germany), which was freshly calibrated before each measurement campaign. O_2 concentrations after purging solutions with N_2 and before initiating the H_2O_2 transformation

to O_2 were typically between 1.5-5 µM (Figure S2a, were used for blank correction) and reached up to 100 µM after addition of HOCl and ascorbic acid (Figure S2b). Blank corrected O_2 concentrations of the transformed samples from ozonation of acrylic acid, cinnamic acid, phenol and sorbic acid are summarised in Figure S3.

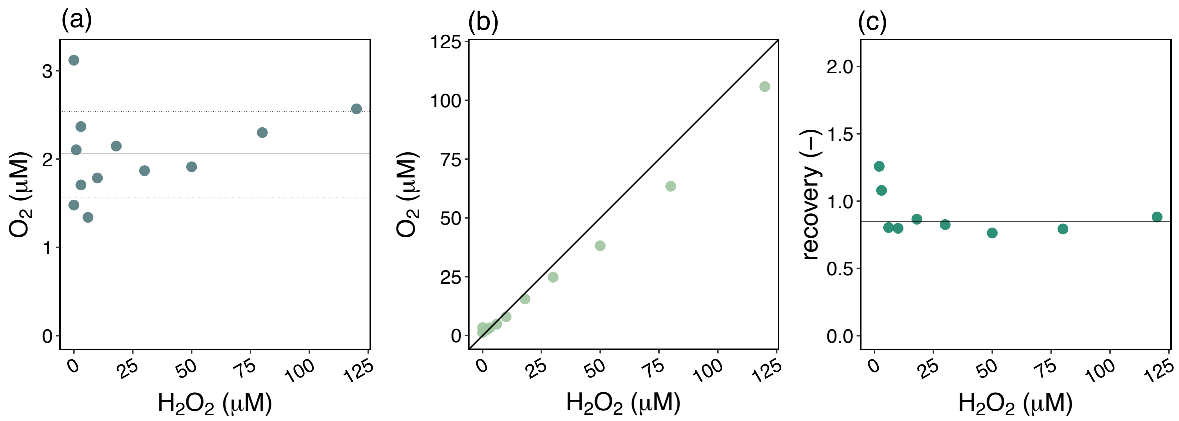

Figure S2. O_2 measurements by a needle-type oxygen microsensor (NTH-PSt7): (a) O_2 blanks, (b) calibration curve (range 0 – 120 µM transformed H_2O_2 in ultrapurified water) which is transformed to O_2 by HOCl (1:1 line in black), (c) recovery of O_2 from transformed H_2O_2.

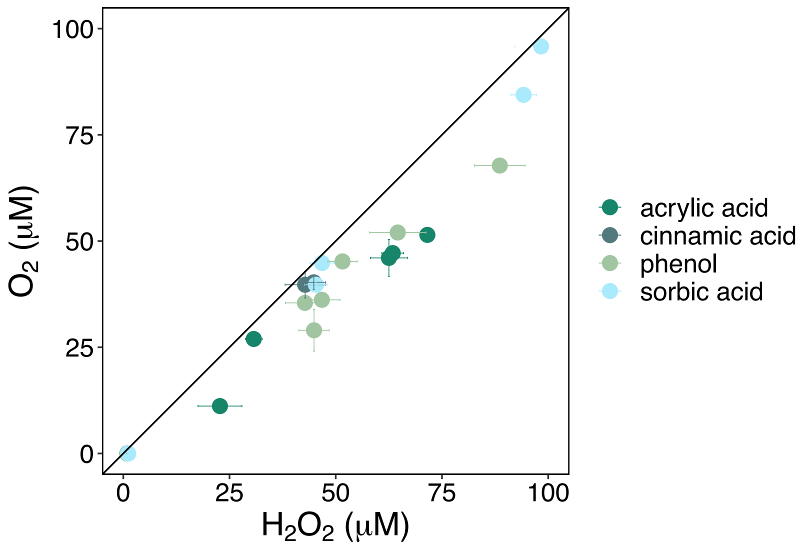

Figure S3. H_2O_2 concentrations from the ozonation of olefins and phenol vs. corresponding measured O_2 concentration (measured with a needle-type oxygen microsensor (NTH-PSt7)) after transformation of H_2O_2 by HOCl. The line represents a 1:1 molar ratio.

S3.3 Oxygen isotope ratio measurements by GC/IRMS

Instrumental analysis mostly followed procedures developed by Bopp et al., 2022 and procedural descriptions are reproduced here with some modifications. Gaseous and headspace

samples in 21 mL crimp vials were analysed by gas chromatography (GC)/IRMS consisting of a GC coupled via a Conflo IV interface to a Delta V Plus isotope ratio mass spectrometer (Thermo Fisher Scientific, Reinach, Switzerland). All samples were placed on a Combi PAL autosampler (CTC Analytics, Zwingen, Switzerland) for isotopic analysis. The injection was performed using a 2.5 mL gas-tight headspace syringe (HD-type, gauge 23, CTC Analytics). Prior to sample loading, the syringe was flushed with N_2 for 1 min. For each measurement, 1500 μL of gaseous or headspace sample was single injected into the split injector with He as carrier gas (99.999%) and a split flow of 40 mL /min. Chromatographic separation of O_2, N_2 and Ar was achieved by two 30 m Rt-Molsieve 5 A PLOT column (Restek from BGB Analytik; 30 m x 0.32 mm ID, 30 μm film thickness) and a PLOT column particle trap (Restek from BGB Analytik; 2.5 m x 0.32 mm ID).[10,11] The O_2 pulses were introduced into a GC combustion III interface (Thermo Fisher Scientific) equipped with a Nafion membrane to remove water and subsequently into a Delta Plus XL isotope ratio mass spectrometer (Thermo Fisher Scientific). $\delta^{18}O$ values were determined from ratios of peak areas of masses 32 and 34 versus reference gas pulses of O_2 introduced at the beginning of each chromatogram (99.995%, 2.98 ± 0.15 V peak height, σ $\delta^{18}O$ = 0.07 ‰, n =221). The $\delta^{18}O$ value of the reference gas was adjusted to O_2 peaks from on-column injections of ambient air (70 μL) thereby assuming a constant $\delta^{18}O$ of 23.88 ‰.[13]

S3.4 Data evaluation

Evaluation of $^{18}O/^{16}O$ ratio measurements of O_2 followed peak integration and blank correction procedures as described in detail previously.[10,11] In brief, automatic peak detection was performed with Isodat NT 3.0 (Thermo Fisher Scientific) applying time-based background determination 1 min prior to the O_2 peak. Blank correction for diffuse contamination from ambient O_2 were performed with blank (in ultrapurified water) and control samples (in sample matrix), respectively, according to established procedures.[14] The peak areas of blank samples

of the individual GC/IRMS measurements are shown in Figure S4. The peak areas of control samples of the model compound ozonation experiments are shown in Figures S10-S13.

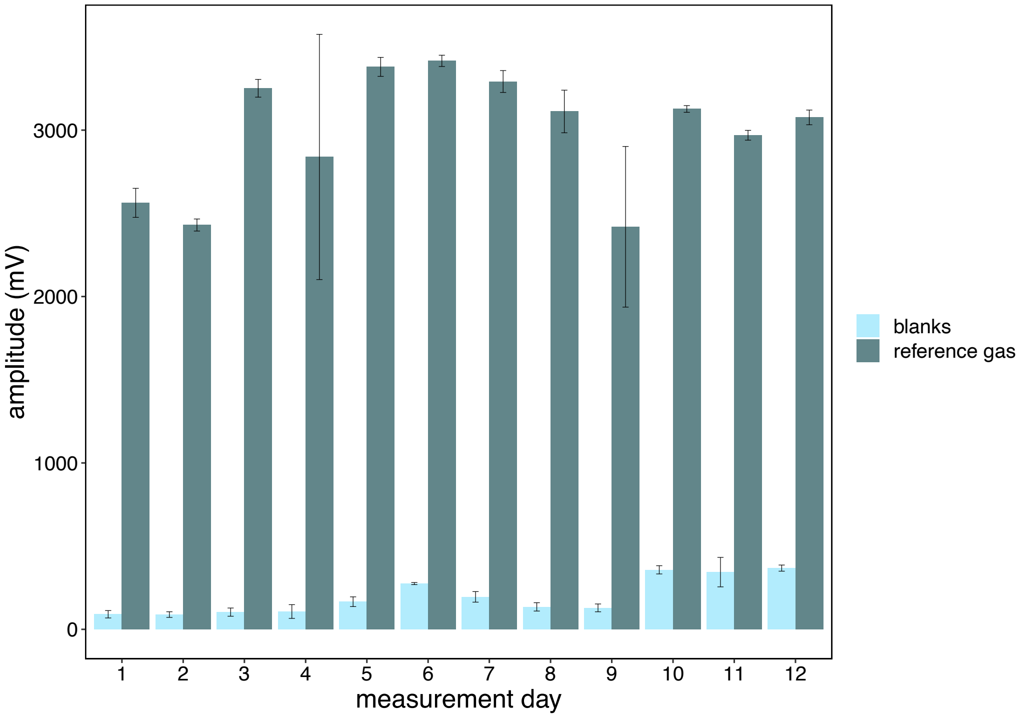

Figure S4. Variation of mean amplitudes of the blanks (light blue) and reference gas (petrol) measured on the GC/IRMS over the course of 12 individual measurement days. After higher variations on measurement day 4 and 9, the gas-tight headspace syringe for injection was replaced. The filament of the GC/IRMS had to be replaced after measurement day 4. $\delta^{18}O$ of blanks and reference gas, respectively: 15.45 ± 2.06 ‰ (n = 52) and 23.88 ± 0.07 ‰ (n=221).

Section S4. Application of isotopic H_2O_2 characterisation to ozonation experiments

S4.1 Conservation of H_2O_2 prior to conversion to O_2

Effects of time, pH and organic peroxides on H_2O_2 concentrations: At room temperature, H_2O_2 very slowly (within several days) disproportionates to H_2O and O_2.[15] Therefore, for the timeframe of the transformation experiment (within 2-3 hours) the disproportionation is negligible. Consequently, H_2O_2 concentrations originating and determined from commercially available stocks are considered stable and reliable for the transformation reactions. However, H_2O_2 which is formed upon ozonation of model

compounds might be influenced by other solution components. One example is the presence of glyoxylic acid, a frequently detected ozonation-induced byproduct (i.e. 100% yield during cinnamic acid ozonation). Glyoxylic acid and H_2O_2 react to formic acid and CO_2 with pH dependent second-order rate constants ranging from ~100 $M^{-1}s^{-1}$ (pH 10) to < 0.3 $M^{-1}s^{-1}$ (below pH 5).[6] Control experiments at pH 3 showed that H_2O_2 was stable over 2 hours (typical time period required for sample processing for conversion to O_2) in contrast to samples at pH 7 (Figure S5a). Consequently, all ozonated model compound solutions were immediately acidified with phosphoric acid to pH 3 to ensure stability of H_2O_2. Furthermore, the presence of organic peroxides can lead to H_2O_2 formation as they are in equilibrium with a carbonyl compound and H_2O_2 (Figure 1, main manuscript). However, typically these rate constants to achieve equilibrium are in the order of 10^{-6} up to 10^{-4} s^{-1},[16] making formation of H_2O_2 within the time frame of the experiments negligible.

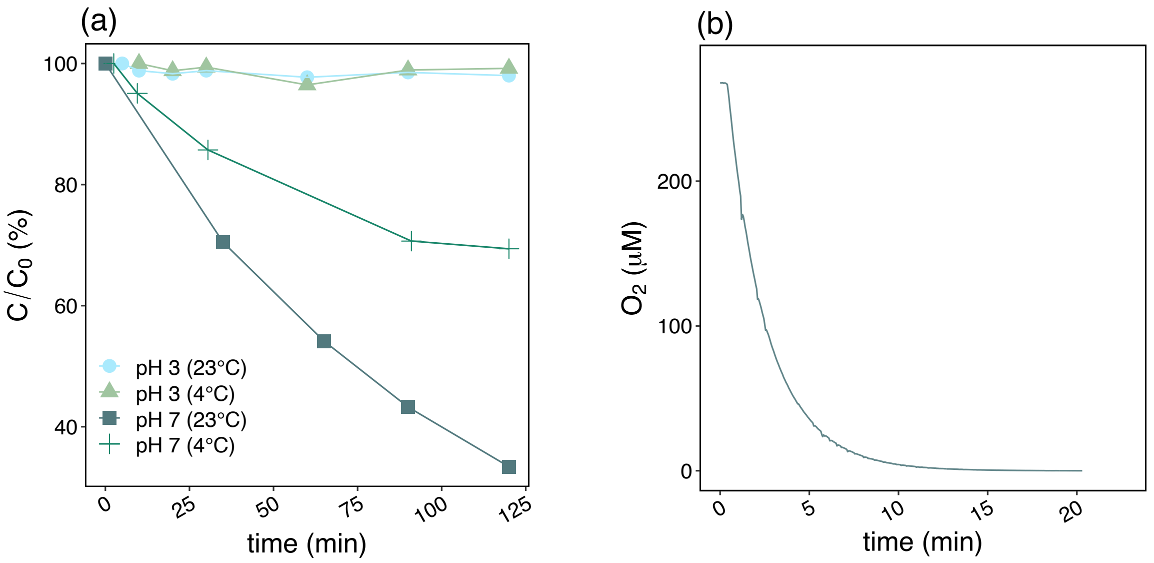

Figure S5. (a) Relative residual H_2O_2 as a function of time and effects of pH and T in solutions with initial concentrations of 500 µM glyoxylic acid, 500 µM H_2O_2 and cinnamic acid (1 mM at pH 7, 0.5 mM at pH 3) and 10 mM phosphate buffer. (b) Measured oxygen removal during purging with N_2 over time of a 110 mL H_2O_2 solution. No duplicate experiments were performed.

Effects of purging time on H_2O_2 concentrations: 10-15 min purging was sufficient to reach O_2 levels < 0.4 ppm for volumes between 80-110 mL (Figure S5b). Purging removes

negligible amounts of H_2O_2 (data not shown). The latter can be explained by Henry's law constant of H_2O_2 which is 7 orders of magnitude higher ($8.3 \cdot 10^2$ $mol \cdot m^{-3} \cdot Pa^{-1}$) compared to O_2 (10^{-5} $mol \cdot m^{-3} \cdot Pa^{-1}$).[17]

Effects of sample transfer on H_2O_2 and O_2 concentrations: The transfer of sample vials into the glovebox was performed immediately after purging solutions with N_2 to remove O_2 and thus it was again assumed that disproportionation of H_2O_2 is negligible. O_2 concentrations are expected to decrease further during sample handling because of the overpressure and continuous flushing with N_2 in the glovebox.

S4.2 Effect of solutes on HOCl-based H_2O_2 conversion to O_2

Effects of pH on conversion efficiency: The rate of the reaction of HOCl with H_2O_2 is higher at higher pH, with a maximum at pH 9.65.[5] However, the pH was always ≤ 7, because otherwise, side reactions may occur. At pH 3, the reaction conditions might not allow a complete transformation of H_2O_2 to O_2 with the applied HOCl dose. Thus, the H_2O_2 conversion to O_2 was always performed at pH 7.

Effects of DMSO on H_2O_2 conversion efficiency: In ozonation experiments, an $^{\bullet}OH$ radical scavenger is typically added to ensure only direct reactions between ozone and the model compound. In the present study, DMSO was used as radical scavenger, which reacts with HOCl with a $k_{HOCl\text{-}DMSO}$ of 315 $M^{-1}s^{-1}$ at pH 7, 20 °C.[18] Thus, higher HOCl concentrations were necessary in the presence of DMSO to maintain the same turnover. The latter is illustrated in Figure S6a, which shows the transformed H_2O_2 after a first (black circles) and a second (red circles) addition of 1 mL HOCl to ~50 µM H_2O_2 solutions as a function of increasing DMSO (NIR-PMT measurements). The doses of HOCl and NaOH to be added to achieve full conversion in the experiments was assessed prior to each H_2O_2 transformation experiment with a control sample and was in the range of 9-20 µL NaOH (5M) and 50-200 µL HOCl (~1.5-1.7 M) for the 21 mL crimp vials.

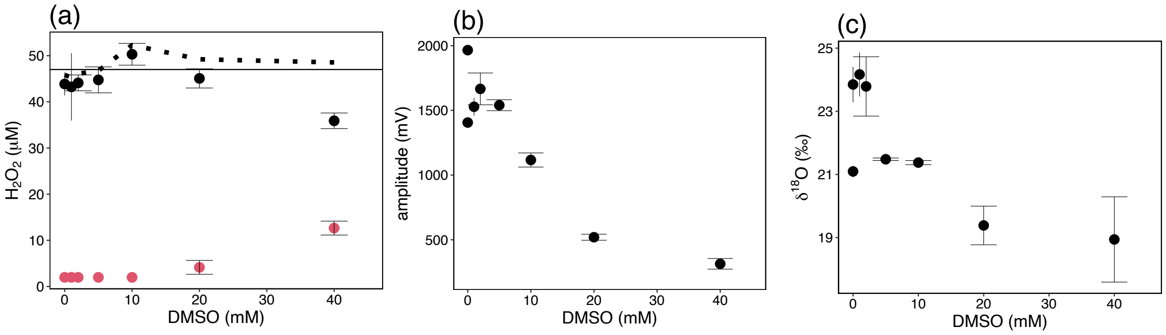

Figure S6. Influence of DMSO on analytical procedure for H_2O_2 in buffered solutions. (a) H_2O_2 concentration (measured as $^1O_2$) as a function of increasing DMSO concentration (black circles). A second HOCl dose in NIR-PMT was necessary to quantify the remaining H_2O_2 (red circles) and achieve a complete mass balance (dotted line). The solid line represents the nominal H_2O_2 concentration. (b) Injected O_2 (amplitudes in mV) detected by the IRMS as a function of increasing DMSO concentration, (c) $\delta^{18}O$ values as a function of increasing DMSO concentration. Amplitudes ranged between 300 and 2000 mV. Experimental conditions: 48 µM H_2O_2, 0-40 mM DMSO, phosphate buffer pH 7, 10 mM.

S4.3 Effects of solution constituents on $^{18}O/^{16}O$ ratio measurements by GC/IRMS

Effects of H_2O_2 conversion efficiency on $^{18}O/^{16}O$ ratio measurements: Figure S6b/c show the influence of DMSO on the conversion efficiency to O_2 and thus the signal amplitudes of O_2 peaks in the IRMS as well as $\delta^{18}O$ values. If insufficient HOCl doses are applied (see discussion previous paragraph) the incomplete H_2O_2 turnover leads to O_2 signal intensities below the method detection limits and thus inaccurate $\delta^{18}O$ values as a function of increasing DMSO concentrations (Figure S6c).

Effects of ascorbic acid and diffusion on O_2 concentrations: O_2 can react slowly with ascorbic acid (the quenching reagent) which would lead to a depletion of O_2 and thus potential isotopic fractionation. However, control measurements did not indicate a relevant decrease in O_2 for isotopic measurement at pH 7. Further, diffusion of O_2 from ambient air into the vials is possible, which would lead to an O_2 increase and influence the $\delta^{18}O$. The latter was monitored using a blank which underwent the same procedure and no substantial increase in O_2 was observed over the measurement sequence (low variation for blanks in Figure S4). O_2

impurities caused by the sample handling (needle injections) were corrected using matrix blanks as described in Pati et al., 2016.

Effects of pH on $^{18}O/^{16}O$ ratio measurements: The importance of a consistent pH, which ensures a complete turnover of H_2O_2 to O_2 is exemplified in Figure S7 with $\delta^{18}O$ values of transformed H_2O_2 from cinnamic acid ozonation at pH 3 with and without pH adjustment to pH 7.

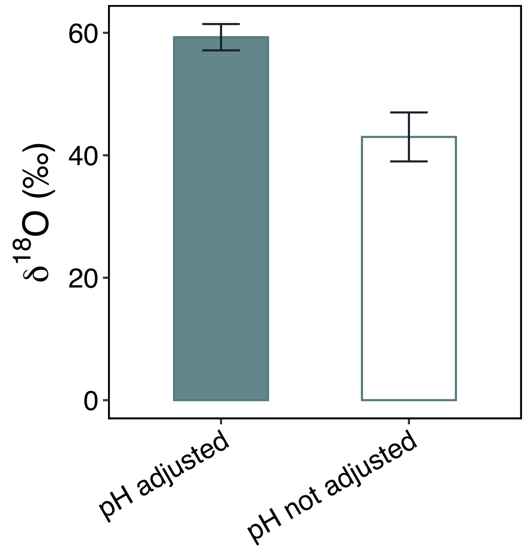

Figure S7. $\delta^{18}O$ values of H_2O_2 from cinnamic acid ozonation at pH 3 with pH adjustment (filled bar) and without pH adjustment (empty bar) to pH 7. Experimental conditions: Ozonation: 200 µM cinnamic acid, 10 mM phosphate buffer pH 3, 5 mM DMSO and O_3 doses of 40 µM. Transformation of H_2O_2 to O_2: dose of 50 µL 1.5 M NaOCl with and without 15 µL 5 M NaOH.

S4.4 Method detection limits for $^{18}O/^{16}O$ ratio measurements in H_2O_2

Figure S8 and S9 show (a) the amplitudes and (b) derivation of the method detection limit (MDL) for the $^{18}O/^{16}O$ ratio measurements in H_2O_2 according to the moving mean procedure.[19] The O isotope ratios can be determined as low as 3 µM in ultrapurified water and 12 µM in sample matrix, respectively, corresponding to signal amplitudes of 376 and 496 mV. With a typical experimental matrix (10 mM phosphate buffer and 5 mM DMSO) a higher MDL is rationalised by higher blanks.

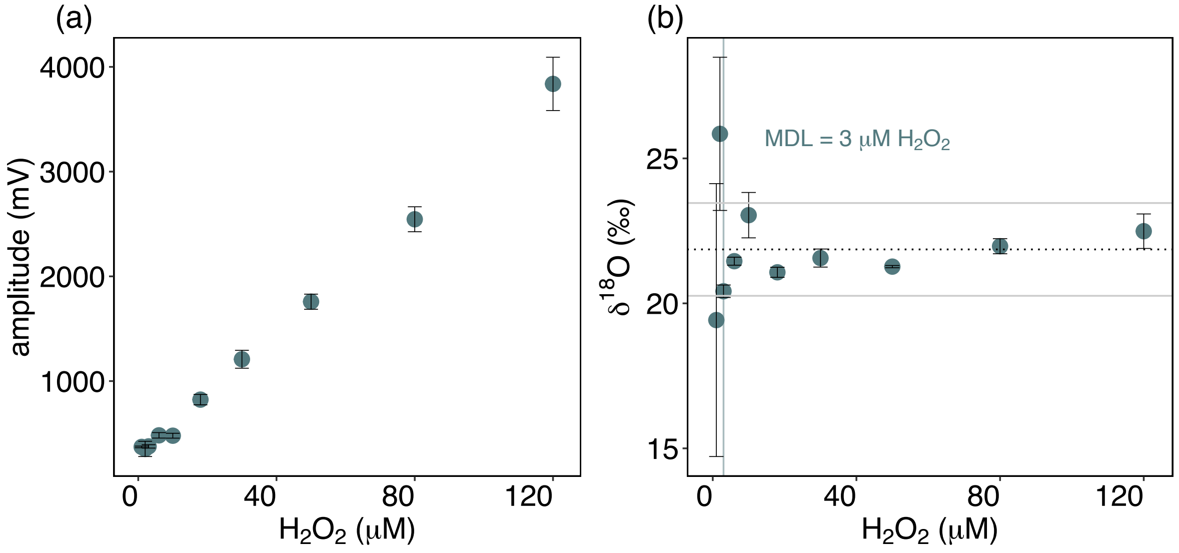

Figure S8. (a) Amplitude test for O_2 from transformed H_2O_2 standard solutions and (b) corresponding $\delta^{18}O$ values as a function of the H_2O_2 concentrations. Black dotted line: mean $\delta^{18}O$. Experimental conditions: ultrapurified water without pH control.

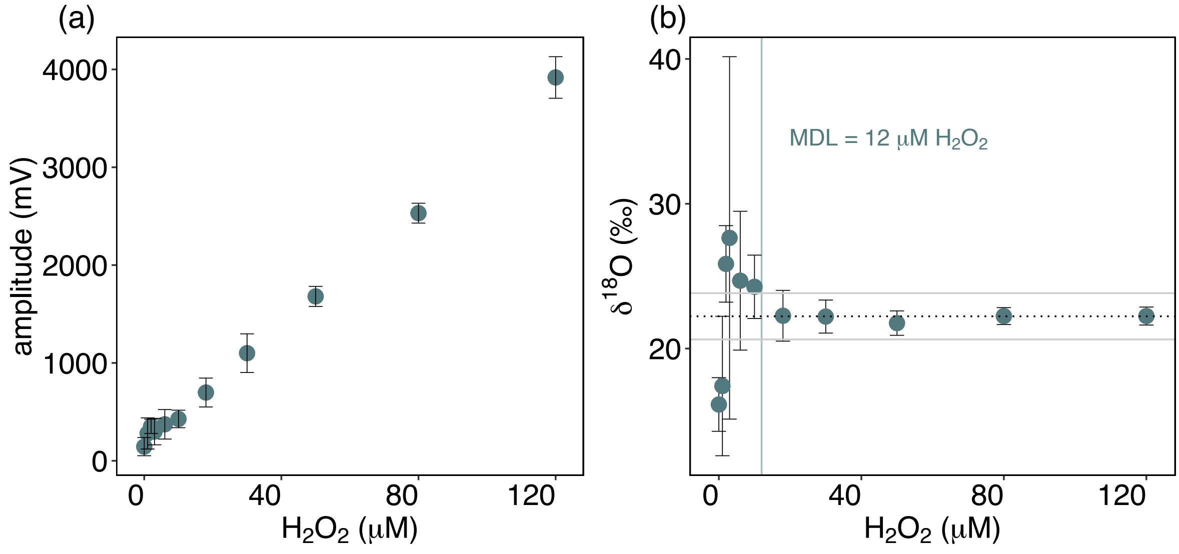

Figure S9. (a) Amplitude test for O_2 from transformed H_2O_2 buffered solutions and (b) corresponding $\delta^{18}O$ values as a function of the H_2O_2 concentrations. Black dotted line: mean $\delta^{18}O$. Experimental conditions: ultrapurified water with pH control, 10 mM phosphate buffer (pH 7) and 5 mM DMSO.

S4.5 Overview of model compound analysis and $^{18}O/^{16}O$ ratio measurements

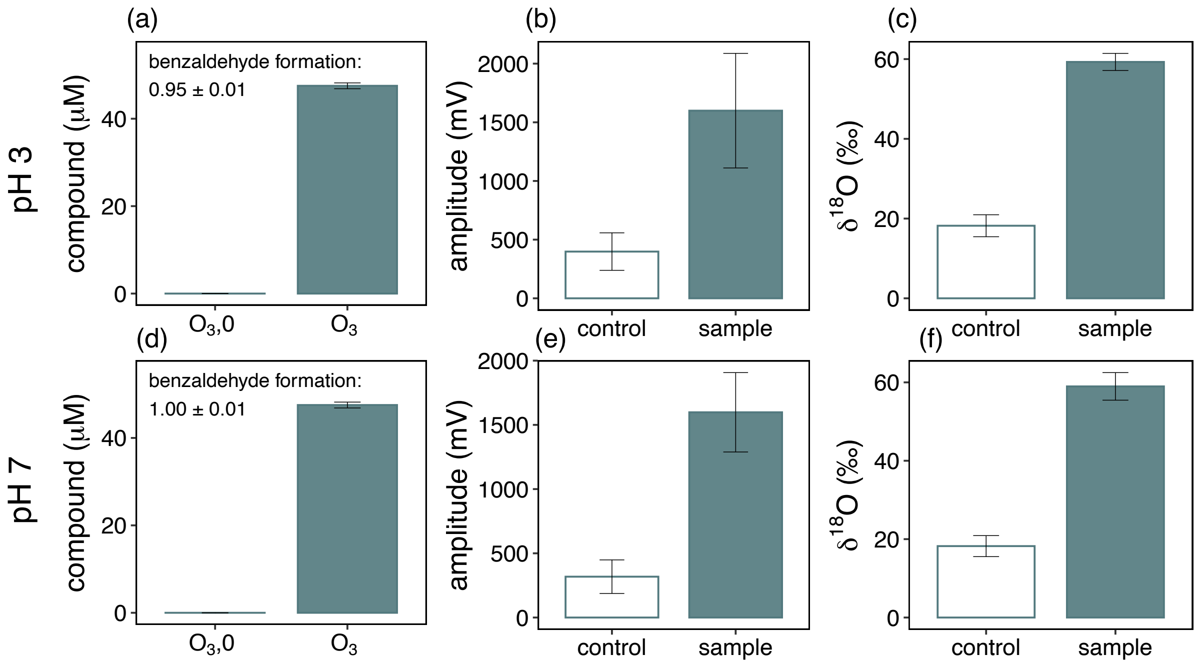

Figure S10. Reactions of ozone with cinnamic acid/cinnamate at pH 3 and 7. (a, d) Formation of benzaldehyde upon ozonation (inserted text: molar yield based on O_3 dose), (b, e) amplitude and (d, f) $\delta^{18}O$ of control and sample of transformed O_2 from H_2O_2. Experimental conditions: 200 µM cinnamic acid, 10 mM phosphate buffer, 5 mM DMSO and O_3 dose 45 µM.

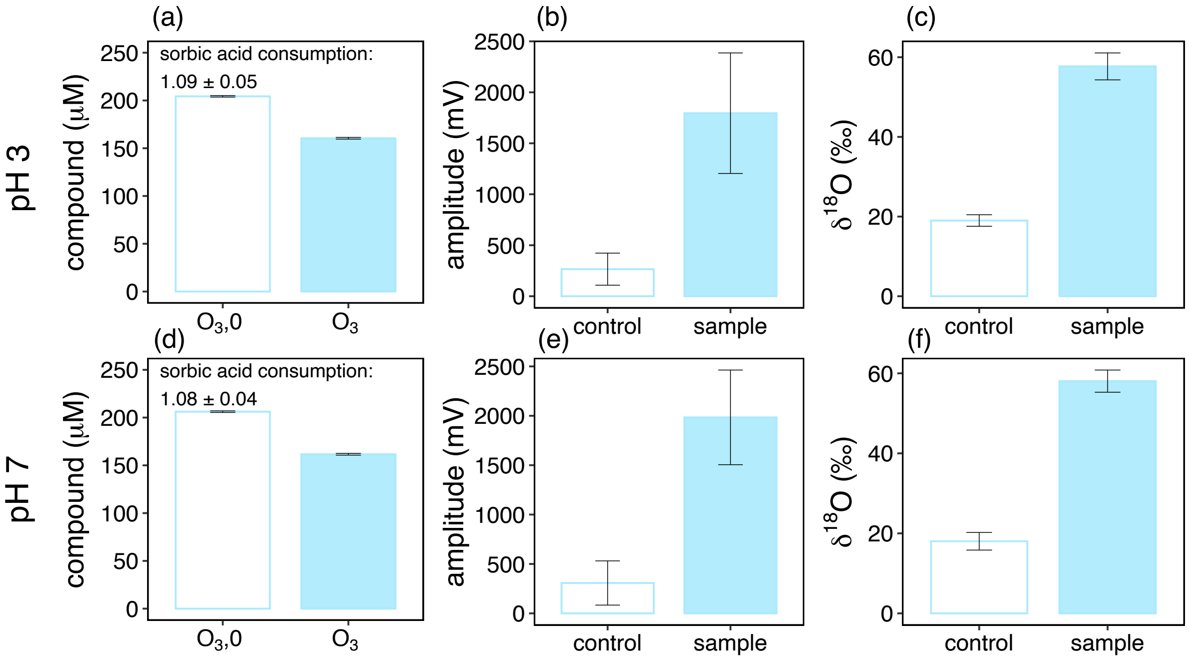

Figure S11. Reaction of ozone with sorbic acid/sorbate at pH 3 and 7. (a, d) consumption of sorbic acid upon ozonation (inserted text: molar consumption based on O_3 dose), (b, e) amplitude and (d, f) $\delta^{18}O$ of control and sample of transformed O_2 from H_2O_2. Experimental conditions: 200 µM sorbic acid, 10 mM phosphate buffer, 5 mM DMSO and O_3 dose 40 µM.

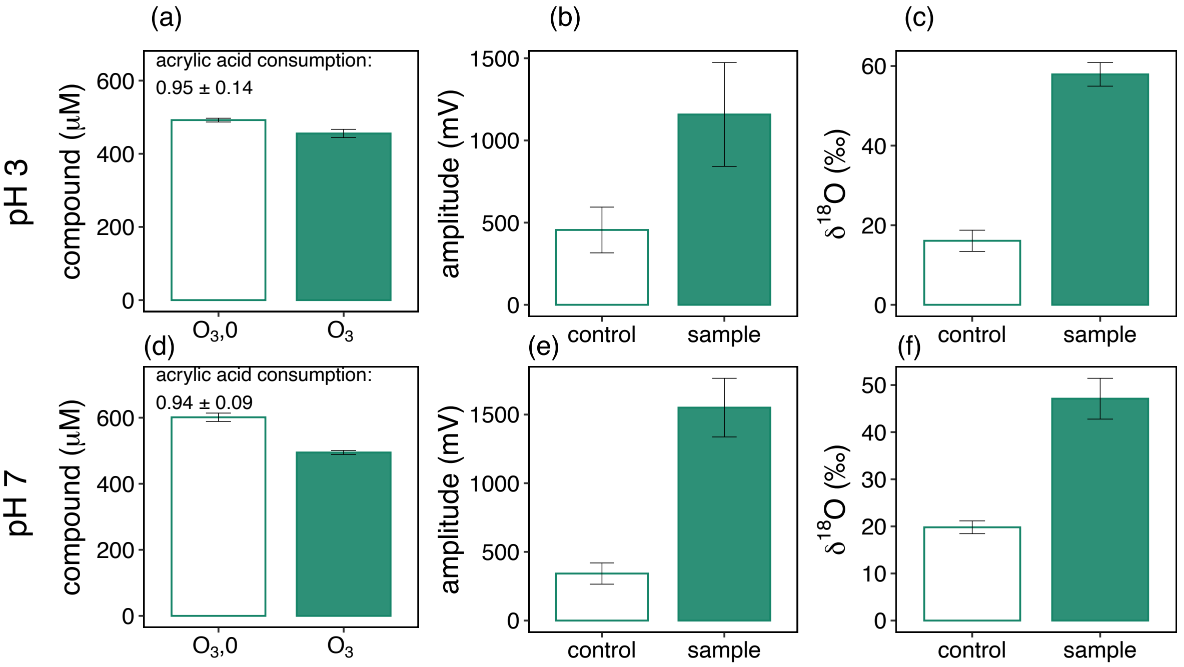

Figure S12. Reaction of ozone with acrylic acid/acrylate at pH 3 and 7. (a, d) consumption of acrylic acid upon ozonation (inserted text: molar consumption based on O_3 dose), (b, e) amplitude and (c, f) $\delta^{18}O$ of control and sample of transformed O_2 from H_2O_2 at pH 3 and 7, respectively. Experimental conditions: 600 μM acrylic acid, 10 mM phosphate buffer, 15 mM DMSO and O_3 doses 120 μM.

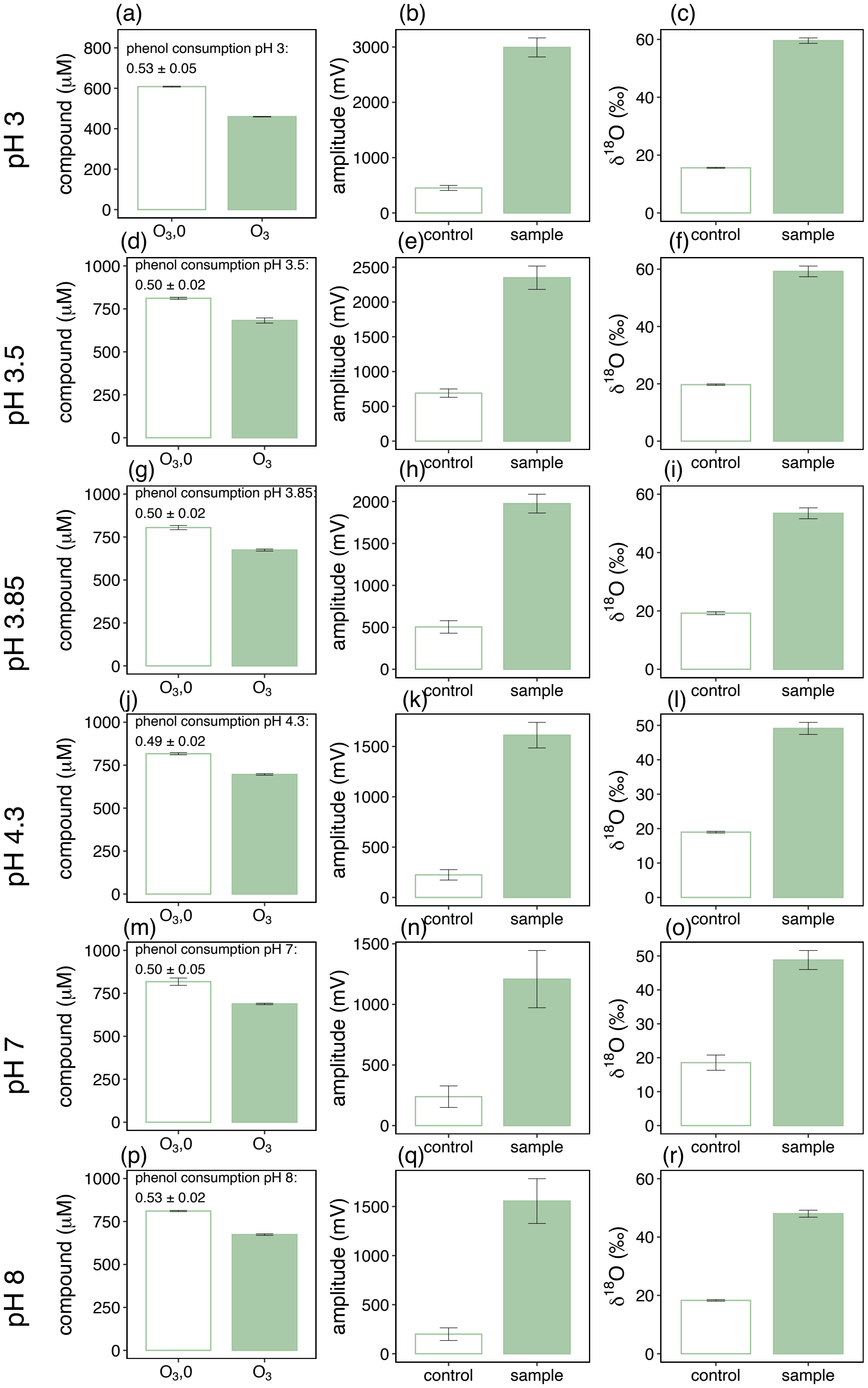

Figure S13. Reaction of ozone with phenol/phenolate at pH 3, 3.5, 3.85, 4.3, 7 and 8. (a, d, g, j, m, p) consumption of phenol upon ozonation (inserted text: molar consumption based on O_3 dose), (b, e, h, k, n, q) amplitude and (c, f, i, l, o, r) $\delta^{18}O$ of control and sample of transformed O_2 from H_2O_2. Experimental conditions: 600 µM phenol (pH 3), 800 µM phenol (pH > 3), 10 mM phosphate buffer, 25 mM DMSO (pH 3), 33 mM DMSO (pH > 3) and O_3 doses of 267 µM.

Table S5. Overview $\delta^{18}O$ of H_2O_2 from the selected model compounds at different pH values (10 mM phosphate buffer).

**Model compound**	**pH**	**$\delta^{18}O$ (‰)**	**Technical replicates**	**Experimental replicates**
Phenol/phenolate	3	59.6 ± 0.9	6	2
Phenol/phenolate	3.5	59.2 ± 1.9	8	2
Phenol/phenolate	3.85	53.5 ± 1.9	8	2
Phenol/phenolate	4.3	49.1 ± 1.8	8	2
Phenolate	7	48.8 ± 2.8	22	8
Phenolate	8	48.0 ± 1.2	7	2
Acrylic acid	3	57.9 ± 3.0	12	4
Acrylate	7	47.1 ± 4.3	13	5
Sorbic acid	3	57.7 ± 3.4	10	4
Sorbate	7	58.1 ± 2.8	10	4
Cinnamic acid	3	59.3 ± 2.1	18	7
Cinnamate	7	59.0 ± 3.5	17	6

Section S5. Approach for the derivation of $\delta^{18}O$ in O_3

$\delta^{18}O$ of O_3 was determined indirectly in a mass-balance approach by measurements of O isotope ratios of O_2 by GC/IRMS in an identical manner as described for transformed H_2O_2. Given that O_3 typically coexists with residual O_2 in aqueous solutions, $\delta^{18}O$ of O_3 ($\delta^{18}O_{O3}$) was derived from the comparison of $\delta^{18}O$ from solutions type (i) containing both O_3 and O_2 ($\delta^{18}O_{O3+O2}$) with $\delta^{18}O$ of solutions type (ii) where O_3 was removed and only the residual O_2 ($\delta^{18}O_{O2}$) was left behind.

Type (i) solution: 1200 µL ultrapurified water at pH 12 (adjusted with 1 M NaOH) was purged for 1 min with N_2 in a 12 mL crimp vial. The vial was filled up with O_3/O_2-containing stock solution (1.91 mM) using a 20 mL Hamilton syringe and under stirring. Additionally, a needle was inserted to the rubber of the crimp cap, to release excess air. Under these basic conditions,

O_3 decays by a chain reaction to O_2 (see eqs. S1-S6 below) and thus the solution will contain O_2 from the carrier gas and O_3 (control).

$O_3 + OH^- \rightarrow HO_4^-$	70 $M^{-1}s^{-1}$	(S1)
$HO_4^- \rightleftarrows HO_2^{\cdot} + O_2^{\cdot -}$	$\Delta G°$=14.8 kJ/mol	(S2)
$HO_2^{\cdot} \rightleftarrows O_2^{\cdot -} + H^+$	p*K*a = 4.8	(S3)
$O_2^{\cdot -} + O_3 \rightarrow O_2 + O_3^{\cdot -}$	$1.5 \cdot 10^9$ $M^{-1}s^{-1}$	(S4)
$O_3^{\cdot -} \rightrightarrows OH^{\cdot} + O_2$	$1.4 \cdot 10^3$ s^{-1}	(S5)
$OH^{\cdot} + O_3 \rightarrow HO_2^{\cdot} + O_2^{\cdot -}$	$1.1 \cdot 10^8$ $M^{-1}s^{-1}$	(S6)

Because $^{\cdot}OH$ lead to a catalytic O_3 decay, it can be assumed, that O_3 will be completely transformed to O_2.

Type (ii) solution: 1200 µL 220 mM cinnamic acid solution at pH 8.5 was purged with N_2 in a 12 mL crimp vial. The vial was filled up with O_3/O_2-containing stock solution (1.91 mM) as described above. In presence of cinnamic acid, ozone is completely depleted and the $\delta^{18}O$ of the residual O_2 from the O_3/O_2 mixture can be determined.

In all samples, a headspace of 3 mL was created with an overpressure of 1.3-1.4 bar in a similar manner as for the H_2O_2 samples. 70 µL were injected into the GC/IRMS.

The necessary parameters and equations for the determination of the $\delta^{18}O$ in O_3 are outlined below:

1. The solubility of a gas is determined by the temperature and its partial pressure:

$$p_{O_3} = H^{cp}\left(T_{\frac{O3}{O2}}\right) \cdot c_{O_3} \quad \text{(S7)}$$

with p_{O_3} as the partial pressure of O_3, and $H^{cp}\left(T_{\frac{O3}{O2}}\right)$ the temperature dependent Henry's law solubility constant (with $T_{\frac{O3}{O2}}$ as temperature of the O_3/O_2-containing solution) and c_{O_3} as the concentration of O_3 in the O_3/O_2-containing solution (eqs. below).

2. The temperature dependent Henry's law solubility constant is determined with measured parameters and literature values;[17] T_{RT} = 298.15 K, $T_{O3/O2}$ = 275.15 K, p_{atm} = 101 325 Pa, $H^{cp}_{O_3,T_{RT}}$ = 1.0 · 10^{-4} mol/m^{3}Pa, $H^{cp}_{O_2,T_{RT}}$ = 1.3 · 10^{-5} mol/m^{3}Pa parameter for the T dependency of H ($-\Delta_{sol,O3}H/R$) = 2800 K and H ($-\Delta_{sol,O2}H/R$) = 1500 K.

$$H\left(T_{\frac{O3}{O2}}\right) = H^{cp}{}_{T_{RT}} \cdot e^{\left(\frac{-\Delta_{sol}H}{R}\left(\frac{1}{T_{\frac{O3}{O2}}} - \frac{1}{T_{RT}}\right)\right)} \quad \text{(S8)}$$

$$H^{cp}\left(T_{\frac{O3}{O2}}\right) = H\left(T_{\frac{O3}{O2}}\right) \cdot \frac{p_{atm}}{1000} \quad \text{(S9)}$$

With these equations, $H^{cp}\left(T_{\frac{O3}{O2}}\right)$ are 2.2 · 10^{-2} M/atm for O_3 and 2.0 · 10^{-3} M/atm for O_2, respectively.

3. The O_3 concentration was determined experimentally as c_{O3} = 1.91 · 10^{-3} M. Using eq. S7, the partial pressure can be determined as 0.086 atm for O_3. Assuming 1 atm in the equilibrated system, the partial pressure of O_2 has to be $p_{O_2} = 1 - p_{O_3}$ = 1-0.086 = 0.914 atm. Solving eq. S10 for c_{O2}, and implementing $H^{cp}\left(T_{\frac{O3}{O2}}\right)$ of O_2 (eqs. S8 and S9), a concentration of 1.83 · 10^{-3} M is obtained.

$$c_{O_2} = \frac{(1 - p_{O_3})}{H^{cp}\left(T_{\frac{O3}{O2}}\right)} \quad \text{(S10)}$$

4. To determine the $\delta^{18}O$ of O_3 (eq. S12) the fractional concentration of each gas solute ($f_{c,O2} + f_{c,O3} = 1$) in the O_2/O_3-containing solution (c_{tot} = 1.91 · 10^{-3} M + 1.83 · 10^{-3} M) and the $\delta^{18}O$ of the sample type (i) ($\delta^{18}O_{O2+O3}$ = 8.27 ‰, n = 1) and type (ii) ($\delta^{18}O_{O2}$ =

11.5 ± 1.2 ‰, n = 2) have to be taken into account (eq. S11). $f_{c,O2}$ and $f_{c,O3}$ are 0.49 and 0.51, respectively. Solved for $\delta^{18}O$ eq. S12 is obtained.

$$\delta^{18}O_{O_2} \cdot f_{O_2} + \delta^{18}O_{O_3} \cdot f_{O_3} = \delta^{18}O_{O_2+O_3} \quad \text{(S11)}$$

$$\delta^{18}O_{O_3} = \frac{\delta^{18}O_{O_2+O_3} - \delta^{18}O_{O_2} \cdot f_{O_2}}{f_{O_3}} \quad \text{(S12)}$$

$\delta^{18}O_{O2+O3}$ of 8.27 ‰ is very close to the $\delta^{18}O_{ref}$ of 8.35 ± 0.04 ‰ (n=2) determined with the pure oxygen used for ozone production. With eq. S12, the $\delta^{18}O$ in O_3 can be calculated as 5 ± 1 ‰. A pressure of 1 atm is only an assumption. However, increasing or decreasing it by a factor of 2 decreases or increases the $\delta^{18}O$ of O_3 to 2‰ or 7‰, respectively, and thus has no major influence.

The $\delta^{18}O$ in O_3 is in contrast to the isotopically enriched signature of O_3 observed in the Earths atmospheric O_3, which ranges between a $\delta^{18}O$ of 75-155 ‰.[15] However, O_3 used in this study was produced from O_2 gas (8.35 ± 0.04 ‰) using an ozone generator by a plasma in a microdischarge column, where the enrichment of light isotopes makes sense considering the lower energy barrier to cleave ^{16}O–^{16}O compared to ^{16}O–^{18}O bonds on O_2.[20]

Section S6. Background information to explain isotopic signatures

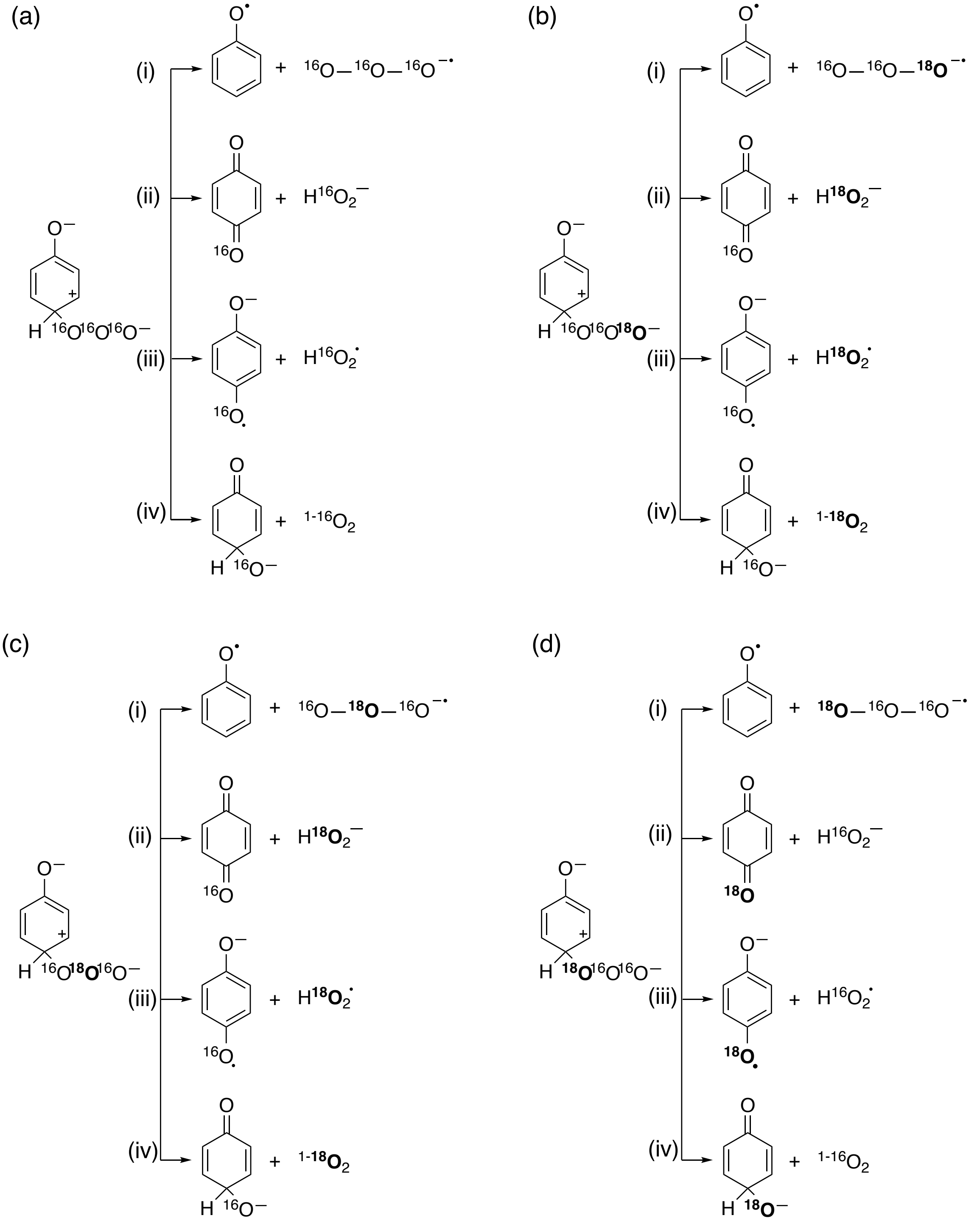

Figure S14. Isotopologues and isotopomers of the ozone-adduct (a-d) with ensuing formation of (i) phenoxyl radical and ozone radical anion, (ii) benzoquinone, HO_2^-; (iii) benzoquinone radical and peroxyl radical ($HO_2^•$) and (iv) precursor of hydroquinone and singlet oxygen ($^1O_2$).

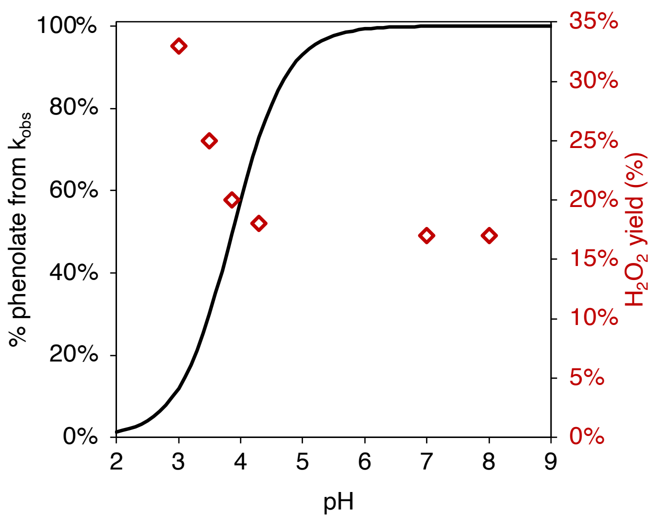

Figure S15. Calculated fraction of phenolate contributing to k_{obs}, the apparent second-order rate constant for the reaction of phenol with O_3 (black line, k_{phenol} = $1.5 \cdot 10^3$ $M^{-1}s^{-1}$ and $k_{phenolate}$= $1.8 \cdot 10^6$ $M^{-1}s^{-1}$ were used to obtain k_{obs})[21] and yield of H_2O_2 (per mole of O_3 consumed) during the ozonolysis of phenol (diamonds) as a function of pH. Experimental conditions: 600 µM phenol (pH 3), 800 µM phenol (pH > 3), 10 mM phosphate buffer, 25 mM DMSO (pH 3), 33 mM DMSO (pH > 3) and O_3 doses of 267 µM.

References

(1) Bader, H.; Hoigné, J. Determination of Ozone in Water by the Indigo Method. *Water Res* **1981**, *15* (4), 449–456. https://doi.org/10.1016/0043-1354(81)90054-3.

(2) von Sonntag, C.; von Gunten, U. *Chemistry of Ozone in Water and Wastewater Treatment-From Basic Principles to Applications*; IWA publishing, 2012.

(3) Buxton, G. v; Greenstock, C. L.; Phillips Helman, W.; Ross, A. B.; Helman, W. P. Critical Review of Rate Constants for Reactions of Hydrated Electrons, Hydrogen Atoms and Hydroxyl Radicals (·OH/·O−) in Aqueous Solution. *J Phys Chem Ref Data* **1988**, *17* (2), 513–886. https://doi.org/10.1063/1.555805.

(4) Pryor, W. A.; Giamalva, D. H.; Church, D. F. Advances in Modem Environmental Toxicology. *Gaseous Air Pollutants and Plant Metabolism* **1984**, *106* (2), 173–183.

(5) Lim, S.; McArdell, C. S.; von Gunten, U. Reactions of Aliphatic Amines with Ozone: Kinetics and Mechanisms. *Water Res* **2019**, *157*, 514–528. https://doi.org/10.1016/j.watres.2019.03.089.

(6) Leitzke, A.; Reisz, E.; Flyunt, R.; von Sonntag, C. The Reactions of Ozone with Cinnamic Acids: Formation and Decay of 2-Hydroperoxy-2-Hydroxyacetic Acid. *Journal of the Chemical Society, Perkin Transactions 2* **2001**, *0* (5), 793–797. https://doi.org/10.1039/b009327k.

(7) Leitzke, A.; von Sonntag, C. Ozonolysis of Unsaturated Acids in Aqueous Solution: Acrylic, Methacrylic, Maleic, Fumaric and Muconic Acids. *Ozone Sci Eng* **2009**, *31* (4), 301–308. https://doi.org/10.1080/01919510903041354.

(8) Tentscher, P. R.; Bourgin, M.; von Gunten, U. Ozonation of Para -Substituted Phenolic Compounds Yields p -Benzoquinones, Other Cyclic α,β-Unsaturated Ketones, and Substituted Catechols. *Environ Sci Technol* **2018**, *52* (8), 4763–4773. https://doi.org/10.1021/acs.est.8b00011.

(9) Mvula, E.; von Sonntag, C. Ozonolysis of Phenols in Aqueous Solution. *Org Biomol Chem* **2003**, *1* (10), 1749. https://doi.org/10.1039/b301824p.

(10) Bopp, C. E.; Bolotin, J.; Pati, S. G.; Hofstetter, T. B. Managing Argon Interference during Measurements of 18O/16O Ratios in O2 by Continuous-Flow Isotope Ratio Mass Spectrometry. *Anal Bioanal Chem* **2022**, *414* (20), 6177–6186. https://doi.org/10.1007/s00216-022-04184-3.

(11) Pati, S. G.; Bolotin, J.; Brennwald, M. S.; Kohler, H.-P. E.; Werner, R. A.; Hofstetter, T. B. Measurement of Oxygen Isotope Ratios (18O/ 16O) of Aqueous O2 in Small Samples by Gas Chromatography/Isotope Ratio Mass Spectrometry. *Rapid Communications in Mass Spectrometry* **2016**, *30* (6), 684–690. https://doi.org/10.1002/rcm.7481.

(12) Pati, S. G.; Kohler, H.-P. E.; Hofstetter, T. B. Characterization of Substrate, Cosubstrate, and Product Isotope Effects Associated With Enzymatic Oxygenations of Organic

Compounds Based on Compound-Specific Isotope Analysis. *Methods Enzymol* **2017**, *596*, 291–329. https://doi.org/10.1016/BS.MIE.2017.06.044.

(13) Barkan, E.; Luz, B. High-Precision Measurements of 17O/16O and 18O/16O of O2 and O2/Ar Ratio in Air. *Rapid Communications in Mass Spectrometry* **2003**, *17* (24), 2809–2814. https://doi.org/10.1002/rcm.1267.

(14) Werner, R. A.; Brand, W. A. Referencing Strategies and Techniques in Stable Isotope Ratio Analysis. *Rapid Communications in Mass Spectrometry*. John Wiley & Sons, Ltd April 15, 2001, pp 501–519. https://doi.org/10.1002/rcm.258.

(15) Guo, H.; Yu, X.; Lin, M. Kinetic Isotope Effects in H2O2 Self-Decomposition: Implications for Triple Oxygen Isotope Systematics of Secondary Minerals in the Solar System. *Earth Planet Sci Lett* **2022**, *594*, 117722. https://doi.org/10.1016/j.epsl.2022.117722.

(16) Dowideit, P.; von Sonntag, C. Reaction of Ozone with Ethene and Its Methyl- and Chlorine-Substituted Derivatives in Aqueous Solution. *Environ Sci Technol* **1998**, *32* (8), 1112–1119. https://doi.org/10.1021/es971044j.

(17) Sander, R. Compilation of Henry's Law Constants (Version 4.0) for Water as Solvent. *Atmos Chem Phys* **2015**, *15* (8), 4399–4981. https://doi.org/10.5194/acp-15-4399-2015.

(18) Amels, P.; Elias, H.; Wannowius, K. J. Kinetics and Mechanism of the Oxidation of Dimethyl Sulfide by Hydroperoxides in Aqueous Medium: Study on the Potential Contribution of Liquid-Phase Oxidation of Dimethyl Sulfide in the Atmosphere. *Journal of the Chemical Society - Faraday Transactions* **1997**, *93* (15), 2537–2544. https://doi.org/10.1039/a700722a.

(19) Jochmann, M. A.; Blessing, M.; Haderlein, S. B.; Schmidt, T. C. A New Approach to Determine Method Detection Limits for Compound-Specific Isotope Analysis of

Volatile Organic Compounds. *Rapid Communications in Mass Spectrometry* **2006**, *20* (24), 3639–3648. https://doi.org/10.1002/rcm.2784.

(20) Willi, A. v. *Isotopeneffekte Bei Chemischen Reaktionen*; Thieme: Stuttgart, 1983.

(21) Hoigné, J.; Bader, H. Rate Constants of Reactions of Ozone with Organic and Inorganic Compounds in Water—II: Dissociating Organic Compounds. *Water Res* **1983**, *17* (2), 185–194. https://doi.org/10.1016/0043-1354(83)90099-4.
